# Sulfur metabolism in subtropical marine mangrove sediments fundamentally differs from other habitats as revealed by SMDB

**DOI:** 10.1038/s41598-023-34995-y

**Published:** 2023-05-19

**Authors:** Shuming Mo, Bing Yan, Tingwei Gao, Jinhui Li, Muhammad Kashif, Jingjing Song, Lirong Bai, Dahui Yu, Jianping Liao, Chengjian Jiang

**Affiliations:** 1 National Engineering Research Center for Non-Food Biorefinery, Guangxi Research Center for Biological Science and Technology, Guangxi Academy of Sciences, Nanning, 530007 China; 2 State Key Laboratory for Conservation and Utilization of Subtropical Agro-bioresources, Guangxi Research Center for Microbial and Enzyme Engineering Technology, College of Life Science and Technology, Guangxi University, Nanning, 530004 China; 3 Guangxi Key Lab of Mangrove Conservation and Utilization, Guangxi Mangrove Research Center, Guangxi Academy of Sciences, Beihai, 536000 China; 4 Guangxi Key Lab of Human-Machine Interaction and Intelligent Decision, Nanning Normal University, Nanning, 530299 China; 5 State Key Laboratory of Radiation Medicine and Protection, Soochow University, Suzhou, 215123 China; 6 Guangxi Key Laboratory of Beibu Gulf Marine Biodiversity Conservation, Beibu Gulf University, Qinzhou, 535011 China

**Keywords:** Microbiology, Biogeochemistry, Environmental sciences

## Abstract

Shotgun metagenome sequencing provides the opportunity to recover underexplored rare populations and identify difficult-to-elucidate biochemical pathways. However, information on sulfur genes, including their sequences, is scattered in public databases. Here, we introduce SMDB (https://smdb.gxu.edu.cn/)—a manually curated database of sulfur genes based on an in-depth review of the scientific literature and orthology database. The SMDB contained a total of 175 genes and covered 11 sulfur metabolism processes with 395,737 representative sequences affiliated with 110 phyla and 2340 genera of bacteria/archaea. The SMDB was applied to characterize the sulfur cycle from five habitats and compared the microbial diversity of mangrove sediments with that of other habitats. The structure and composition of microorganism communities and sulfur genes were significantly different among the five habitats. Our results show that microorganism alpha diversity in mangrove sediments was significantly higher than in other habitats. Genes involved in dissimilatory sulfate reduction were abundant in subtropical marine mangroves and deep-sea sediments. The neutral community model results showed that microbial dispersal was higher in the marine mangrove ecosystem than in others habitats. The *Flavilitoribacter* of sulfur-metabolizing microorganism becomes a reliable biomarker in the five habitats. SMDB will assist researchers to analyze genes of sulfur cycle from the metagenomic efficiently.

## Introduction

Microorganisms play essential roles in sulfur cycle, which determine the compounds of sulfur transformation and their fate in the environment^1^. Sulfur compounds are abundant in natural environments, and a huge storage of sulfate and sulfides is found in marine ecosystems^2^. The sulfur cycle, mainly driven by sulfur oxidation and sulfate reduction, is tightly intertwined with other biochemical cycles (i.e., carbon, nitrogen, phosphorus) with far-reaching implications for environmental ecosystem^3^. Based on previous reports, sulfate-reducing bacteria (SRB) play a crucial role in the precipitation of heavy metals^4^, pollutants^5^, and hydrocarbon biodegradation^6^. Thus, characterizing the sulfur cycling genes and sulfur-metabolizing microorganisms is important to understand sulfur cycling processes in the environments.

The sulfur cycle is a complex biochemical process in the environment, consisting of inorganic and organic sulfur transformations. Inorganic transformations (i.e., canonical dissimilatory sulfate reduction [DSR], and assimilatory sulfate reduction [ASR]) have been well studied as described in the previous study^7^. For example, the composition of microorganism communities showed that Deltaproteobacteria was the dominant class of SRB and the pathway of ASR was major sulfate reduction in a full-scale biofilm-membrane bioreactor for textile wastewater treatment^7^. Organic sulfur transformations have a significant role in the sulfur cycle, given the abundance of organic sulfur in the environmental ecosystem^8^. Previous research has focused on inorganic sulfur transformations, hence the impact of organic sulfur compounds on ecosystems has yet to be explored^3^. Organosulfur compounds were abundant in the marine environment, such as dimethylsulfoniopropionate (DMSP)^9^, sulfonates^3^, sulfate esters^3^, and methanethiol (MeSH)^10^. The DMSP enzymatic breakdown product (dimethyl sulfide [DMS]) may result in global warming^9^. Sulfonates are decisive ecological prevalence for energy interchange between microbial autotrophs and heterotrophs, indicating the importance of organic sulfur metabolism in the environment^11^. Thus, it is critical to developing capabilities to obtain the complete sulfur cycle via advanced technologies.

Previously, considerable effort has been made to characterize sulfur cycle processes by analyzing key genes, such as dissimilatory sulfite reductase (*dsrB*)^12^, adenylyl sulfate reductase (*aprA*)^13^, and thiosulfohydrolase (*soxB*)^14^. Given the need for suitable DNA primers for many sulfur genes, polymerase chain reaction (PCR) usually produces inaccurate experimental results^15,16^. However, shotgun metagenome sequencing provides the opportunity to recover underexplored sulfur cycle^17^. Potential genes involved in sulfur cycling were annotated for metagenomics analysis using orthology database^18^. However, a comprehensive and reliable orthology database is essential for accurate annotation of functional genes. Thus, the results of the metagenomic analysis are heavily influenced by the selection of orthology databases.

Given the unavailability of a comprehensive sulfur database, multiple databases, such as Clusters of Orthologous Groups (COG)^19^, Kyoto Encyclopedia of Genes and Genomes (KEGG)^20^, Evolutionary Genealogy of Genes: Non-supervised Orthologous Groups (eggNOG)^21^, and M5nr^22^, are widely used for the efficient metagenomic data set. However, a database with high coverage of sulfur genes is further required^23^. Given the similar functional domains of the genes, small error abundance values from the application of these databases in analyzing sulfur cycles should be avoided^24^. Searching sulfur cycling genes in large databases is time consuming. Therefore, a specific database of genes related to the sulfur cycle pathway should be developed to address these problems.

The microbial community assembly processes determine species distribution patterns and abundance^25^. The neutral theory posits that stochastic processes (dispersal, local extinction, and ecological drift) cause variation in microbial community composition^26^. According to this theory, sulfur cycle in different areas may present distinct due to geographical distance. Mangroves are widely distributed along tropical and subtropical coasts to withstand waves and storms. Recent studies have investigated sulfur metabolism in mangrove ecosystems^27^. However, it is still being determined how microbial communities and the sulfur cycle differ from other biomes, such as upland forests, deep-sea sediments, marine waters, and freshwater.

A manually integrated database, namely, the sulfur metabolism gene integrative database (SMDB), gathering most of the sulfur cycling genes from public databases (i.e., KEGG, COG, eggNOG, M5nr, and NR), has been developed to address the limitations of currently available public resources and facilitate the identification and characterization of sulfur genes and sulfur-metabolizing microorganism communities. Our motivations in creating SMDB are as follows: (i) to improve the sulfur pathway about functional genes and associated microorganisms and (ii) to facilitate the annotation of sulfur cycle information in shotgun sequencing. SMDB was applied to functionally and taxonomically identify sulfur cycling genes and sulfur-metabolizing microorganism communities from different habitats (upland forest, deep-sea sediments, marine waters, river sediments, and mangrove sediments). This study could provide a high-quality sulfur cycle database for functional profiling of shotgun metagenomes.

## Materials and methods

### SMDB sources

The universal protein (UniProt) database (http://www.uniprot.org/; October 2019) was utilized to retrieve core database sequences of sulfur cycling genes by using keywords. COG (ftp://ftp.ncbi.nih.gov/pub/COG/COG2014/data), eggNOG (http://eggnogdb.embl.de/download/eggnog_4.5/), KEGG (http://www.genome.jp/kegg/; October 2019), and M5nr (ftp://ftp.metagenomics.anl.gov/data/M5nr/current/M5nr.gz; October 2019) were used for retrieving nontarget homologous sequences. In addition, the homologous sequences from the NCBI nonredundant (NR) database (ftp://ftp.ncbi.nlm.nih.gov/blast/db/; October 2019) were added to the core constructed database.

### SMDB development

**Figure 1:**
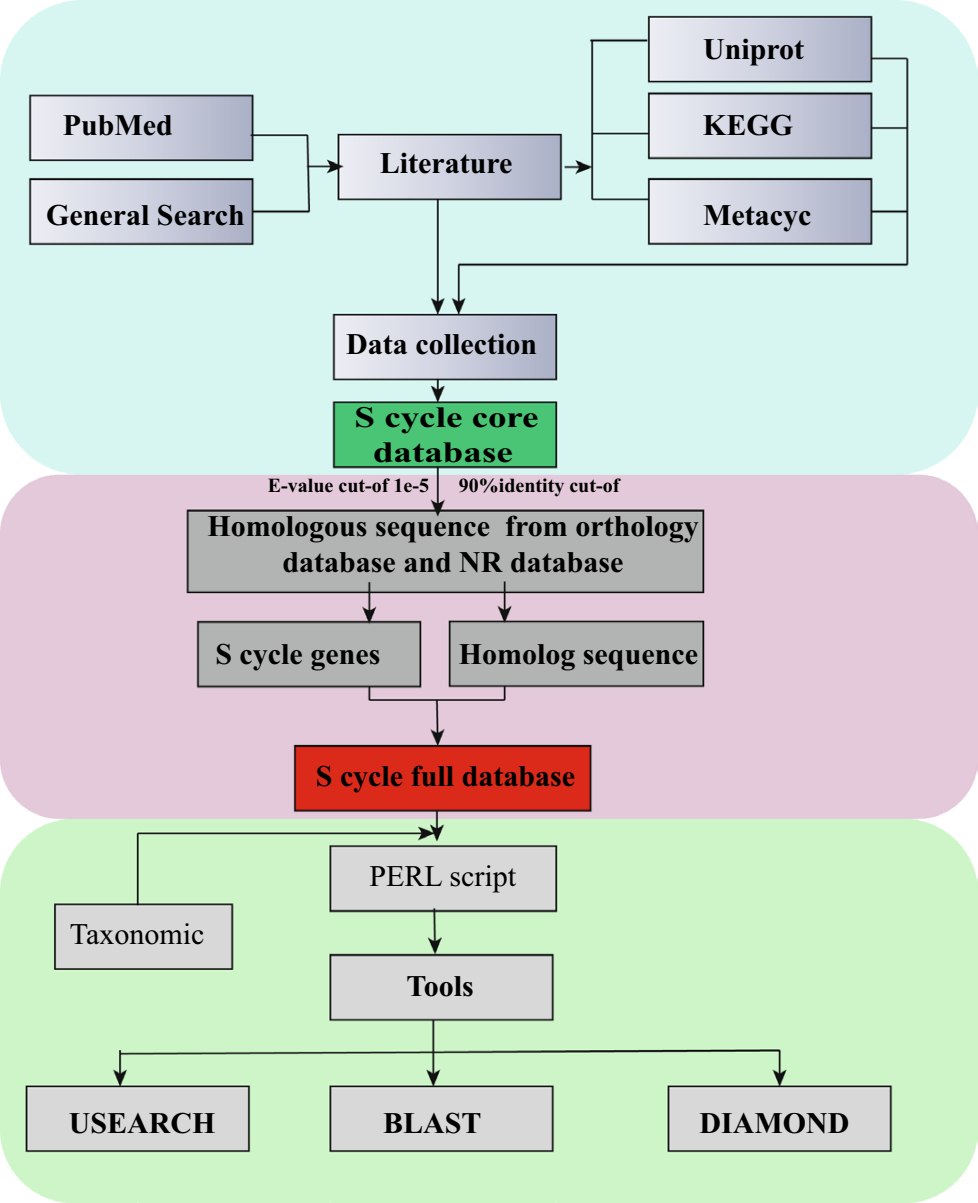
Flowchart of major steps for SMDB construction. First, a core database was constructed for selected genes by retrieving protein sequences from UniProt databases using keywords. Second, a full database was constructed by integrating target genes from databases, including COG, eggNOG, KEGG, M5nr, and NR. Third, a PERL script was developed to generate functional and taxonomic profiles for shotgun metagenomes using searching tools.

An integrated database was manually created to profile sulfur cycling genes from shotgun metagenomes as described by a previous study^28^, with slight modifications. An integrative database of nitrogen cycling genes was manually curated by researchers^28^. The detailed method for database development is described following (Fig. 1).

#### Core database for constructing sulfur cycling genes

The sulfur metabolism in KEGG and MetaCyc databases^29^ were referenced to retrieve genes involved in the sulfur cycle. Only those genes that had been experimentally confirmed to be involved in sulfur metabolism were collected through an extensive literature search. The next step was followed by searching the UniProt database with their keywords (e.g., dissimilatory sulfite reductase alpha subunit: *dsrA*) to download the corresponding annotated sulfur metabolism gene sequences. For genes with vague definitions (e.g., *cysQ* and *MET17*), the full protein name was added in the keywords to exclude the sequences with vague annotations. Then, to ensure the accuracy of SMDB, these sequences for each sulfur cycling gene were manually checked based on their annotations. Finally, these collected sequences were selected as the core database for sulfur cycling genes.

#### Full SMDB construction

COG, eggNOG, KEGG, M5nr, and NR databases were used to retrieve sulfur genes' homologous sequences to construct a complete database and reduce false positives. The integrative database included the core database of sulfur cycling genes and their homologous sequences. The sequence files of this integrative database were clustered using the CD-HIT (v.4.5.6, identity set as 100%)^30^. Representative sequences from this integrative database were subsequently selected to build the SMDB. The sulfur metabolism in KEGG, MetaCyc, and references was referenced to the gene assignments of SMDB sulfur pathways.

For the SMDB taxonomic annotation, the SMDB sequences were aligned to the NR database via BLASTP of DIAMOND (v.0.9.29.130, coverage > 50%, *e*−value < 1 × 10^−10^)^31^. Then, the BLASTP result was performed using the MEGAN software LCA algorithm to get taxonomic classification^32^. The SMDB (v.1) was deposited in GitHub (https://github.com/taylor19891213/sulfur-metabolism-gene-database) on January 8, 2020. In contrast, the analytic platform of the SMDB website has been online since June 22, 2020 (https://smdb.gxu.edu.cn/).

### Implementation of the SMDB in environmental samples

#### Metagenomic analysis

The SMDB was applied to analyze sulfur cycling genes and sulfur-metabolizing microorganism communities from five distinct habitats: upland forest^33^, deep-sea sediments^34,35^, marine waters^36^, river sediments^37^, and mangrove sediments^38^. These data were obtained from the NCBI (https://www.ncbi.nlm.nih.gov/sra) and the Chinese National Genomics Data Center GSA database (https://bigd.big.ac.cn/gsub/) (Supplementary Table S1). The sequence data were merged and assembled by megahit (v1.1.3) with default parameters^39^. The assembled sequences were used for gene prediction by Prodigal (v3.02)^40^. The number of reads in the gene alignment in all samples was calculated by SoapAligner (v2.21)^41^. The gene-normalized abundance was calculated based on the number of reads and gene length. Then, the assembled sequences were annotated using the SMDB, COG, eggNOG, KEGG, M5nr, SCycDB, and NR databases via the DIAMOND with parameters set as an *e*-value cutoff of 1 × 10^−5^^31^. Sulfur sequences are extracted from the merged sequences for further taxonomy annotation. Microorganisms at different taxonomic levels were generated after least common ancestor (LCA) algorithm^42^.

#### Statistical analysis

Shannon indices reflect the diversity of species in the samples^43^. The Shannon indices was calculated by using R package. Principal coordinates analysis (PCoA) was used to describe the differences in microbial community and sulfur gene structure among samples from different regions by using the R package “stats.” The R and P of PCoA are calculated based on ANOSIM using the R package “vegan.” The neutral community model (NCM) was used to explain the microbial community assembly in different habitats^44^. Statistical tests of genes involved in sulfur metabolism between marine and non-marine ecosystems were performed by comparing their abundance by the Tukey–Kramer test. The least significant difference (LSD) test^45^ was used to analyze the variance (ANOVA) model for multiple comparisons among five habitats for sulfur-metabolizing microorganisms.

## Results

### Summary of genes and pathways in SMDB

Using keywords (e.g., sulfur, sulfate) to retrieve 284,541 literature reports from 1976 to 2021 in Web of Science and then obtained records of sulfur genes through a web crawler with Python. After manual verification, 875 related literature reports (representative literature was recruited) and 175 genes covering 11 sulfur metabolism pathways (including assimilatory sulfate reduction, thiosulfate disproportionation, sulfide oxidation, dissimilatory sulfate reduction, sulfite oxidation, sulfur oxidation, sulfur reduction, tetrathionate oxidation, tetrathionate reduction, thiosulfate oxidation, and organic degradation/synthesis), were recruited in the SMDB (Table 1, Supplementary Table S2). Each sulfur gene has rich information, including the mechanism of action, structure, and sequence. The SMDB obtained 395,737 representative sequences at 100% identity cutoffs. Summary of the sulfur metabolism pathway genes see Supplementary Materials.

**Table 1:** Summary of sulfur cycle genes in SMDB and other databases.

Gene	Annotation	Core database sequences	Full database sequences	Other database				
				NR	M5nr	KEGG	eggNOG	COG
*APA1_2*	Sulfate adenylyltransferase (ADP)/ATP adenylyltransferase	3	9	6	0	0	0	0
*APR*	Adenylyl-sulfate reductase (glutathione)	9	152	69	36	0	38	0
*aprA*	Adenylylsulfate reductase, subunit A	151	3769	2483	1041	30	50	14
*aprB*	Adenylylsulfate reductase, subunit B	53	959	517	182	81	104	22
*aprM*	Adenylylsulfate reductase membrane anchor	4	33	10	8	3	8	0
*aps*	Sulfate adenylyltransferase	35	371	172	80	0	84	0
*apt*	Adenylylsulfate:phosphate adenylyltransferase	1	6	4	1	0	0	0
*asrA*	Anaerobic sulfite reductase subunit A	798	2979	1499	423	125	126	8
*asrB*	Anaerobic sulfite reductase subunit B	799	2849	1359	378	138	157	18
*asrC*	Anaerobic sulfite reductase subunit C	415	2234	1175	363	146	117	18
*ATCYSC1*	l-3-cyanoalanine synthase/ cysteine synthase	4	58	29	10	0	15	0
*atsA*	Arylsulfatase	2660	21,439	14,005	3094	817	768	95
*atsK*	Alpha-ketoglutarate-dependent sulfate ester dioxygenase	23	366	271	45	5	22	0
*BPNT1*	3′(2′),5′-bisphosphate nucleotidase	165	925	503	168	0	89	0
*comC*	R-sulfolactate oxidoreductase, comC	4	31	9	6	6	4	2
*comD*	Sulfopyruvate decarboxylase subunit alpha	180	675	317	78	36	52	12
*comE*	Sulfopyruvate decarboxylase subunit beta	163	519	231	47	31	35	12
*cuyA*	l-cysteate sulfo-lyase	1	39	23	4	2	7	2
*cysC*	Adenylylsulfate kinase	16,251	38,287	15,562	3098	1433	1636	307
*cysD*	Sulfate adenylyltransferase subunit 2	10,249	18,083	5633	1045	438	621	97
*cysE*	cysteine synthase	11,057	42,608	20,639	5322	2740	2492	358
*cysH*	Phosphoadenosine phosphosulfate reductase	7392	27,424	14,130	2929	1462	1312	199
*cysI*	Sulfite reductase (NADPH) hemoprotein beta-component	4716	18,377	9782	2031	945	780	123
*cysJ*	Sulfite reductase (NADPH) flavoprotein alpha-component	3530	23,717	15,032	3045	1285	704	121
*cysK*	Cysteine synthase	1462	27,195	17,066	4219	2388	1781	279
*cysN*	Sulfate adenylyltransferase subunit 1	9800	21,823	8823	1576	642	855	127
*cysNC*	Bifunctional enzyme CysN/CysC	1034	2571	1286	111	47	85	8
*cysO*	Cysteine synthase/O-phosphoserine sulfhydrylase/cystathionine beta-synthase	1	8	3	1	1	1	1
*cysQ*	3′(2′),5′-bisphosphate nucleotidase	25,425	51,246	18,616	3460	1645	1826	274
*dddD*	Dimethylsulfoniopropanoate:acyl-CoA transferase, dddD	1	6	1	1	1	1	0
*dddL*	Dimethylpropiothetin dethiomethylase	159	344	156	15	3	11	1
*dddP*	Dimethylsulfoniopropionate lyase	73	677	511	45	7	38	3
*dddQ*	Dimethylsulfoniopropionate lyase	18	107	75	6	2	5	1
*dddW*	Dimethlysulfonioproprionate lyase DddW	20	75	40	7	3	4	1
*dddY*	Dimethylsulfoniopropionate lyase	5	31	22	2	0	2	0
*ddhA*	Dimethylsulfide dehydrogenase subunit alpha	29	220	149	25	7	8	2
*ddhB*	Dimethylsulfide dehydrogenase subunit beta	6	49	22	14	3	3	1
*ddhC*	Dimethylsulfide dehydrogenase subunit gamma	5	21	11	2	1	2	0
*dmdA*	Dimethylsulfoniopropionate demethylase	511	1122	487	71	9	39	5
*dmdB*	3-(methylthio)propionyl-CoA ligase	91	2799	2179	299	113	112	5
*dmdC*	3-(methylthio)propanoyl-CoA dehydrogenase	1	6	1	1	1	1	1
*dmdD*	(Methylthio)acryloyl-CoA hydratase	1	10	4	2	1	1	1
*dmoA*	Dimethyl-sulfide monooxygenase	3	540	461	53	6	17	0
*dmoB*	Dissimilatory dimethyl-sulfide monooxygenase	1	4	2	1	0	0	0
*dmsA*	Anaerobic dimethyl sulfoxide reductase subunit A	1374	24,534	17,993	3848	873	358	88
*dmsB*	Anaerobic dimethyl sulfoxide reductase subunit B	3	3002	2293	368	266	60	12
*dmsC*	Anaerobic dimethyl sulfoxide reductase subunit C	796	9945	6922	1470	576	147	34
*doxA*	Thiosulfate dehydrogenase [quinone] small subunit	2	9	3	2	1	1	0
*doxD*	Thiosulfate dehydrogenase [quinone] large subunit	12	385	227	72	65	9	0
*dsoB*	DMS oxygenase β subunit	1	11	6	3	0	1	0
*dsoC*	DMS oxygenase γ subunit	1	42	28	8	0	5	0
*dsoD*	DMS oxygenase component	1	303	209	75	4	14	0
*dsoE*	DMS oxygenase ε subunit	1	11	6	3	0	1	0
*dsoF*	DMSO monooxygenase reductase component	1	17	9	5	0	2	0
*dsrA*	Dissimilatory sulfite reductase alpha subunit	5278	16,480	6390	4589	73	109	41
*dsrB*	Dissimilatory sulfite reductase beta subunit	6696	19,954	8582	4458	76	108	34
*dsrC*	Dissimilatory sulfite reductase subunit c	4	9	3	2	0	0	0
*dsrD*	Dissimilatory sulfite reductase subunit D (DsrD)	1	3	1	1	0	0	0
*dsrE*	Sulfurtransferase	1	40	17	8	4	7	3
*dsrF*	Sulfurtransferase	603	2762	1567	302	142	126	22
*dsrH*	Sulfurtransferase	1	7	2	1	1	1	1
*dsrJ*	Sulfite reduction-associated complex DsrMKJOP multiheme protein DsrJ	41	115	39	17	7	11	0
*dsrK*	Sulfite reduction-associated complex DsrMKJOP multiheme protein DsrK	75	341	142	51	19	43	11
*dsrM*	Sulfite reduction-associated complex DsrMKJOP protein DsrM	68	187	75	23	5	14	2
*dsrN*	Dissimilatory sulfite reductase subunit N	1	6	1	1	1	1	1
*dsrO*	Sulfite reduction-associated complex DsrMKJOP iron-sulfur protein DsrO	63	133	47	11	3	8	1
*dsrP*	Sulfite reduction-associated complex DsrMKJOP protein DsrP	71	205	86	21	8	15	4
*ETHE1*	Sulfur dioxygenase	75	511	254	99	4	78	1
*fccA*	Cytochrome subunit of sulfide dehydrogenase	86	342	176	46	13	15	6
*fccB*	Sulfide dehydrogenase [flavocytochrome c] flavoprotein chain	502	5276	3638	597	318	185	36
*fsr*	Coenzyme F420-dependent sulfite reductase	3	16	3	2	4	3	1
*glpE*	Thiosulfate sulfurtransferase	9311	27,591	12,065	2945	1895	1221	154
*hdrA*	Heterodisulfide reductase subunit A	335	1480	641	216	134	102	52
*hdrB*	Heterodisulfide reductase subunit B	4	163	61	53	7	36	2
*hdrC*	Heterodisulfide reductase subunit C	293	1127	453	129	125	84	43
*hdrD*	Heterodisulfide reductase iron-sulfur subunit D	142	509	235	52	39	22	19
*hdrE*	Heterodisulfide reductase subunit E	23	106	51	12	9	5	6
*hdrF*	Heterodisulfide reductase, cytochrome reductase subunit	1	4	3	0	0	0	0
*HINT4*	Sulfate adenylyltransferase (ADP)/adenylylsulfatase	1	18	9	3	0	5	0
*hydA*	Sulfhydrogenase subunit alpha	7	37	18	3	4	3	2
*hydB*	Sulfhydrogenase subunit beta (sulfur reductase)	9	68	31	8	10	8	2
*hydD*	Sulfhydrogenase subunit delta	8	59	27	7	8	6	3
*hydG*	Sulfhydrogenase subunit gamma (sulfur reductase)	26	201	92	27	27	21	8
*IMPAD1*	Inositol monophosphatase 3	86	563	277	114	0	86	0
*mccA*	Dissimilatory sulfite reductase MccA	3	18	7	2	2	3	1
*mccB*	Cystathionine gamma-lyase/homocysteine desulfhydrase	96	1833	1198	257	183	86	13
*mddA*	Methanethiol S-methyltransferase	6	152	83	16	40	6	1
*MET10*	Sulfite reductase [NADPH] flavoprotein component	16	146	93	24	5	8	0
*MET17*	O-acetylhomoserine/O-acetylserine sulfhydrylase	3	104	54	44	3	0	0
*MET22*	3'(2'),5'-bisphosphate nucleotidase	75	290	145	38	11	21	0
*MET3*	Sulfate adenylyltransferase	852	1061	118	34	16	40	1
*MET5*	Sulfite reductase subunit beta	17	128	80	21	2	8	0
*metB*	Cystathionine gamma-synthase	2239	23,670	15,207	3360	1495	1220	149
*MPST*	3-mercaptopyruvate sulfurtransferase	3	3	0	0	0	0	0
*msmA*	Methanesulfonate monooxygenase subunit alpha	33	148	84	13	7	11	0
*msmB*	Methanesulfonate monooxygenase subunit beta	6	19	8	2	1	1	1
*msuD*	Methanesulfonate monooxygenase	292	3118	2192	324	124	180	6
*mtsA*	Methylthiol:coenzyme M methyltransferase	29	78	37	5	4	2	1
*mtsB*	Methylated-thiol–corrinoid protein	2	13	5	3	2	1	0
*npsr*	NADH‐dependent persulfide reductase [flavoprotein/rhodanase]	1	15	10	1	1	2	0
*nrnA*	Bifunctional oligoribonuclease and PAP phosphatase NrnA	4431	18,124	9477	2240	964	895	117
*otr*	Octaheme tetrathionate reductase	3	58	33	11	8	3	0
*PAPSS*	3'-phosphoadenosine 5'-phosphosulfate synthase	246	1847	1055	349	0	197	0
*phsA*	Thiosulfate reductase/polysulfide reductase chain A	4	8	4	0	0	0	0
*phsB*	Thiosulfate reductase electron transport protein	623	2236	1021	343	153	72	24
*phsC*	Thiosulfate reductase cytochrome b subunit	67	1377	916	235	133	20	6
*pspE*	Thiosulfate sulfurtransferase	1121	6429	3573	886	490	320	39
*psrA*	Polysulfide reductase chain A	11	279	208	53	1	6	0
*psrB*	Polysulfide reductase chain B	4	33	10	6	6	4	3
*psrC*	Polysulfide reductase chain C	3	28	11	5	4	3	2
*qmoA*	Adenylylsulfate reductase-associated electron transfer protein QmoA	1	7	3	1	1	1	0
*RDL*	Sulfurtransferase	6	42	22	10	2	2	0
*rdlA*	Rhodanese	7	29	15	7	0	0	0
*rhd*	Rhodanese	2	29	15	5	3	3	1
*SAL*	3′(2′),5′-bisphosphate nucleotidase/inositol polyphosphate 1-phosphatase	7	101	47	17	0	30	0
*sat*	Sulfate adenylyltransferase	20,431	51,745	21,785	4434	2118	2606	371
*sdo*	Sulfur dioxygenase	1	9	4	2	1	1	0
*SELENBP1*	Methanethiol oxidase	33	429	223	108	0	65	0
*sfnG*	Dimethylsulfone monooxygenase	19	544	405	73	22	23	2
*sgpA*	Sulfur globule protein	1	5	1	1	1	1	0
*sgpB*	Sulfur globule protein	1	1	0	0	0	0	0
*sgpC*	Sulfur globule protein	1	1	0	0	0	0	0
*shyB*	Sulfhydrogenase II β subunit	12	28	12	1	2	1	0
*shyC*	Sulfhydrogenase II γ subunit	35	81	38	2	3	1	2
*sir*	Sulfite reductase (ferredoxin)	1309	13,463	9048	1649	631	758	68
*sirA*	Sulfite reductase (ferredoxin)	56	513	282	86	39	45	5
*slcC*	S-sulfolactate dehydrogenase	220	1441	973	143	42	54	9
*slcD*	sulfolactate dehydrogenase	2	17	3	4	3	3	2
*SoeA*	Sulfite dehydrogenase (quinone) subunit SoeA	1	19	5	5	2	4	2
*SoeB*	Sulfite dehydrogenase (quinone) subunit SoeB	3	42	21	6	3	6	3
*soeC*	Sulfite dehydrogenase (quinone) subunit SoeC	2	12	3	2	2	2	1
*sor*	Sulfur oxygenase/reductase	15	74	26	21	5	6	1
*sorA*	Sulfite dehydrogenase	289	1605	973	134	67	113	29
*sorB*	Sulfite dehydrogenase	200	934	533	92	39	62	8
*sorT*	Sulfite dehydrogenase	2	63	26	25	1	9	0
*SOX*	Sulfite oxidase Sox	20	156	81	24	3	27	1
*soxA*	l-cysteine S-thiosulfotransferase	789	2632	1358	215	89	154	27
*soxB*	Thiosulfohydrolase	224	1848	1021	322	110	128	43
*SoxC*	Sulfane dehydrogenase subunit SoxC	206	1256	816	105	43	74	12
*soxD*	Sulfur dehydrogenase subunit SoxD	49	401	179	72	43	42	16
*soxL*	Sulfur transferase, periplasm	1	6	1	1	1	1	1
*soxS*	Sulfur/thiosulfate oxidation protein SoxS	2	6	2	2	0	0	0
*soxV*	Sulfur/thiosulfate oxidation protein SoxV	2	6	2	2	0	0	0
*soxW*	Sulfur/thiosulfate oxidation protein SoxW	2	6	2	2	0	0	0
*soxX*	Sulfur oxidation protein SoxX	313	1922	860	314	177	200	58
*soxY*	Sulfur-oxidizing protein SoxY	664	2984	1861	230	65	152	12
*soxZ*	Sulfur-oxidizing protein SoxZ	254	968	573	59	23	59	0
*SQOR*	Eukaryotic sulfide quinone oxidoreductase	18	361	178	101	1	63	0
*sqr*	Sulfide:quinone oxidoreductase	195	1341	710	229	97	81	29
*sreA*	Sulfur reductase molybdopterin subunit	7	57	20	9	8	8	5
*sreB*	Sulfur reductase FeS subunit	1	4	2	1	0	0	0
*sreC*	Sulfur reductase membrane anchor	1	4	2	1	0	0	0
*sseA*	Thiosulfate/3-mercaptopyruvate sulfurtransferase	477	9732	6382	1526	791	471	85
*ssuA*	Sulfonate transport system substrate-binding protein	131	7832	5984	977	539	159	42
*ssuB*	Sulfonate transport system ATP-binding protein	127	5631	4115	736	430	193	30
*ssuC*	Sulfonate transport system permease protein	1552	20,416	13,582	2942	1445	792	103
*ssuD*	Alkanesulfonate monooxygenase	12,583	62,941	39,079	5765	2266	2976	272
*ssuE*	FMN reductase	1791	9770	5639	1159	644	484	53
*SUOX*	Sulfite oxidase	118	975	573	168	15	100	1
*suyA*	(2R)-sulfolactate sulfo-lyase subunit alpha	82	388	204	40	20	35	7
*suyB*	(2R)-sulfolactate sulfo-lyase subunit beta	141	1199	749	155	51	88	15
*tauD*	Taurine dioxygenase	7011	36,949	21,645	4311	1992	1798	192
*tetH*	Tetrathionate hydrolase	5	31	12	7	3	3	1
*Tmm*	Trimethylamine monooxygenase	24	81	29	21	3	3	1
*tmoC*	Toluene monooxygenase system ferredoxin subunit	30	98	48	10	2	7	1
*tmoF*	Toluene monooxygenase electron transfer component	2	6	3	1	0	0	0
*tsdA*	Thiosulfate dehydrogenase	29	547	384	67	41	20	6
*tsdB*	Thiosulfate dehydrogenase electron acceptor	9	127	76	18	10	11	3
*TST*	Thiosulfate/3-mercaptopyruvate sulfurtransferase	1	19	14	1	1	1	1
*ttrA*	Tetrathionate reductase subunit A	1460	5477	3132	584	150	131	20
*ttrB*	Tetrathionate reductase subunit B	611	3361	2054	377	147	140	32
*ttrC*	Tetrathionate reductase subunit C	462	2445	1545	287	99	45	7
*tusA*	Sulfur-carrier protein	11,711	30,759	12,360	2816	1967	1655	250
*xsc*	Sulfoacetaldehyde acetyltransferase	1053	4261	2423	388	128	233	36
*ygaP*	Thiosulfate sulfurtransferase YgaP	1	549	362	94	78	11	0

### Characteristics of the SMDB

**Figure 2:**
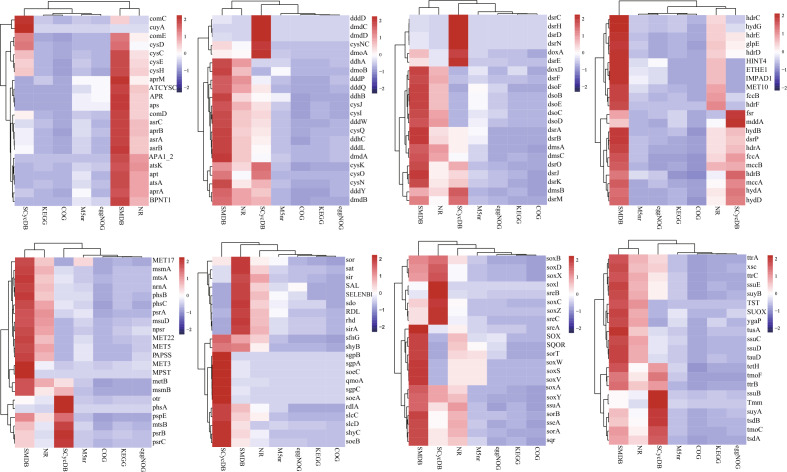
Percentage of sequences belonging to the selected sulfur cycling genes in public databases. Blue indicates fewer gene sequences from the corresponding public database. Heatmap according to the *z*-scores of the abundant gene (sub)family. The heatmap was created using the “pheatmap” package (v1.0.12, https://cran.r-project.org/web/packages/pheatmap/index.html).

The coverage of sulfur metabolism genes in the SMDB was compared with existing public databases to demonstrate the purpose of creating a sulfur metabolism genes database in this study. Among the 175 sulfur genes, we recruited in the SMDB, only 118, 157, 144, 169, and 172 could be found in COG, eggNOG, KEGG, M5nr, and NR databases, respectively (Supplementary Fig. S6). These sequences were counted in public databases and SMDB through Perl programming language for each sulfur gene. The results showed that the coverage of the SMDB containing sulfur gene sequences exceeded that of COG, eggNOG, KEGG, M5nr, and NR databases (Fig. 2). Nearly 342,433 sulfur gene sequences were found in the SMDB and not previously included in NR databases. The 25 metagenomic data obtained from five habitats were aligned against SMDB, COG, eggNOG, KEGG, M5nr, SCycDB, and NR databases to analyze the sulfur metabolism.

### Taxonomic composition of sulfur cycling genes and pathways in SMDB

Sulfur sequences were aligned again with the NR database using a local BLASTP program to obtain the structure and composition of the taxonomic sulfur cycle in SMDB. The SMDB covered 93 phyla, 87 classes, 194 orders, 432 families, and 2225 genera of bacteria, and 17 phyla, 15 classes, 25 orders, 38 families, and 115 genera of archaea (Table 2). For bacteria, Proteobacteria (66.9%), Actinobacteria (14.2%), and Firmicutes (12.1%) were the dominant phyla, with *Pseudomonas* (10.4%), *Escherichia* (5.4%), *Burkholderia* (4.3%), and *Streptomyces* (4.1%) were the dominant genera in the SMDB (Supplementary Table S3). Table 2 shows that assimilatory sulfate reduction has the highest coverage of microorganisms, containing 85 phyla and 1840 genera, followed by organic degradation and synthesis with 54 phyla and 1500 genera and dissimilatory sulfate reduction with 54 phyla and 904 genera. Euryarchaeota, Crenarchaeota, Thaumarchaeota, Candidatus Thorarchaeota, and Candidatus Bathyarchaeota were the dominant phyla of archaea in SMDB (Supplementary Table S3). *Haloferax*, *Haloarcula*, *Archaeoglobus*, *Methanosarcina*, *Thermococcus*, *Nitrosopumilus*, *Methanobrevibacter*, *Methanothrix*, and *Methanobacterium* were the dominant genus of archaea in SMDB (Supplementary Table S3). At the genus level, assimilatory sulfate reduction had the highest diversity involving 79 genera, followed by organic degradation/synthesis (67 genera) and sulfur oxidation (45 genera) (Table 2).

**Table 2:** Summary of sulfur cycle pathways of taxa in SMDB.

Pathway	Phylum		Class		Order		Family		Genus	
	Archaea	Bacteria	Archaea	Bacteria	Archaea	Bacteria	Archaea	Bacteria	Archaea	Bacteria
Assimilatory sulfate reduction	15	85	22	82	22	179	32	403	79	1840
Dissimilatory sulfate reduction	11	54	17	60	17	127	24	267	44	904
Organic degradation and synthesis	7	54	12	70	19	160	25	343	67	1500
Sulfide oxidation	3	15	3	21	4	48	4	86	6	191
Sulfite oxidation	10	53	9	58	17	123	20	263	37	904
Sulfur oxidation	12	45	9	47	15	101	21	249	45	936
Sulfur reduction	9	7	6	12	9	16	12	17	17	20
Tetrathionate oxidation	4	47	6	49	7	104	7	254	9	899
Tetrathionate reduction	5	33	7	44	11	93	11	191	18	518
Thiosulfate disproportionation	3	27	5	39	7	93	7	201	10	545
Thiosulfate oxidation	4	48	6	50	7	106	7	261	10	1043
Total	17	93	15	87	25	194	38	432	115	2225

### Data access and data mining

#### Keyword searches

A simple keyword search was available on the SMDB website, providing a quick means for searching sulfur genes in our database. For example, users interested in dissimilatory sulfite reductase alpha subunit can search for the keyword “*dsrA*”. The search results displayed detailed information about each sulfur gene. An advanced search function allows users to search for pathways of the sulfur cycle.

#### Similarity searches

A DIAMOND interface was also provided to identify and annotate sulfur genes on the SMDB website. 2 G of metagenomic data (i.e., multi-FASTA file and multi-FASTQ file) can be provided to this interface, which consumed 60 s of BLAST time. The web interface tasks required queuing. The output of annotation results was in the standard m8 format; however, additional displays were provided, which were specific to sulfur cycling genes. Our “SMDB annotation format” was inferred from the similarity level to the database sequences associated with a specific sulfur gene.

### Validation

As a specific example, Fun Gene database sequences (i.e., *dsrA/B*, and *soxB*) were used for validation in this study (Table 3). These sequences were annotated using the SMDB via DIAMOND with parameters set as an *e*-value cutoff of 1 × 10^−5^. The results showed that SMDB was able to classify sulfur genes correctly.

**Table 3:** Example database validation.

Gene	Prot_Accno (Fun gene database)	SMDB ID	Identity %	Annotation
*dsrA*	AAC24101.2	49cba574db1e9945c5d3068adf7865cf	100	*dsrA*
	AAB17213.1	NP_069259.1	100	*dsrA*
	AAC24097.2	583ddbe69629b0a6f950bbc94c3a762c	100	*dsrA*
	AAC24103.2	1a6837cbb152f397db0584c361518062	100	*dsrA*
	AAC24105.2	1ea70e110ec9612bc6c8dfb4049c1eb9	100	*dsrA*
*soxB*	YP_865819.1	YP_865819.1	100	*soxB*
	ZP_01748359.1	388399.SSE37_10697	100	*soxB*
	YP_392780.1	326298.Suden_0264	100	*soxB*
	ZP_00961294.1	WP_073034871.1	86.4	*soxB*
	ZP_00963375.1	WP_025055778.1	90.8	*soxB*
*dsrB*	BAB55552.1	WP_092377263.1	100	*dsrB*
	BAB55554.1	33fe083cdd7cc893baaf29a07bde1026	100	*dsrB*
	BAB55556.1	WP_028578006.1	100	*dsrB*
	BAB55558.1	WP_092194129.1	100	*dsrB*
	BAB55560.1	1fc04e092b7119095b7960b78f34cc07	100	*dsrB*

### Implementation of the SMDB for sulfur cycle in environmental samples

#### Microbiome diversity and the community assembly patterns in different habitats

**Figure 3:**
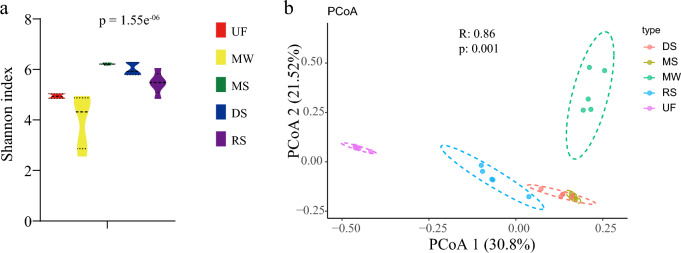
Alpha and beta diversity. (**a**) The α-diversity of microbial diversity for different habitats. ANOVA analyzed significance. (**b**) Principal coordinates analysis (PCoA) of the microbial community. The R and P values in the figure are calculated based on ANOSIM. UF, upland forest; DS, deep-sea sediments; MW, marine waters; RS, river sediments; MS, mangrove sediments.

Overall, 3403 microorganisms were detected from five habitats. Alpha diversity, as measured by the Shannon index, was significantly higher in mangroves than in the other habitats (Fig. 3a). Principle coordinates analysis (PCoA) revealed that microbial beta diversity in five habitats were significantly different (Fig. 3b; ANOSIM, permutations = 999, *p*-value = 0.001, R^2^ = 0.86). The microbial community demonstrated a special composition in mangrove ecosystems, which was predominantly composed of members of Deltaproteobacteria (37.05%), followed by Gammaproteobacteria (28.70%) and Alphaproteobacteria (6.91.8%) (Supplementary Fig. S8). The dominant class in the microbial communities were Alphaproteobacteria (37.59%), followed by Actinomycetia (20.09%) and Acidobacteriia (15.59%) in the upland forest. The dominant class in the microbial communities were Alphaproteobacteria (46.73%), followed by Gammaproteobacteria (21.97%) in marine waters. The dominant class in the microbial communities were Dehalococcoidia (35.15%), followed by Deltaproteobacteria (16.69%) in deep-sea sediments. While, Betaproteobacteria (32.77%) were the dominant class of microbial communities in river sediments (Supplementary Fig. S8).

**Figure 4:**
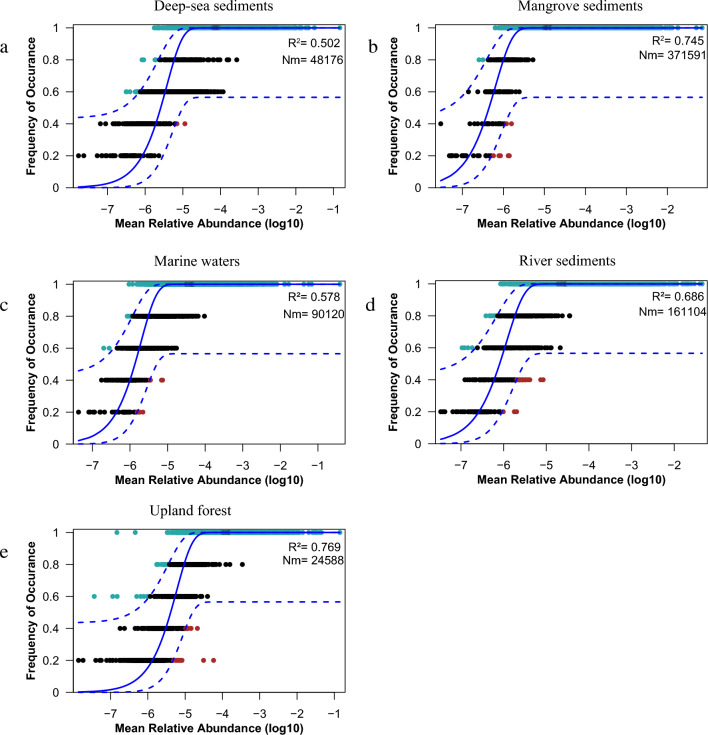
Neutral community model (NCM) of microbial community assembly. (**a**) Deep-sea sediments; (**b**) Mangrove sediments; (**c**) Marine waters; (**d**) River sediments; (**e**) Upland forest. The solid blue lines represent the best fit to the NCM as shown in Sloan et al.^40^, and the dashed blue lines in the figure represent the 95% confidence intervals around the model predictions. Microbial communities with frequencies higher or lower than those predicted by the NCM are shown in different colors. Nm indicates the microbial community size times immigration, and R^2^ represents the fit to this model.

The neutral community model (NCM) fitted well to microbial community assembly in mangrove ecosystems, river sediments, and upland forest habitats (R^2^ > 0.6). However, this model did not fit well with the microbial community assembly in marine waters and deep-sea sediments habitats (Fig. 4). The Nm-value was highest for microbial community in the mangrove ecosystem (Nm = 371,591; Fig. 4), followed by river sediments (Nm = 161,104), marine waters (Nm = 90,120), deep-sea sediments (Nm = 48,176), and upland forest (Nm = 24,588). These results indicated that microbial dispersal was higher in the mangrove ecosystem than in others habitats.

#### Distribution of sulfur genes

**Figure 5:**
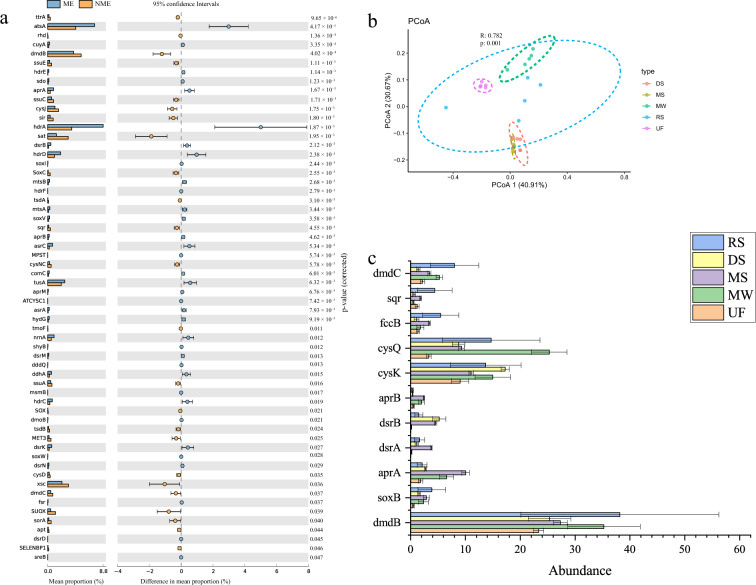
The abundance of sulfur metabolism genes. (**a**) Abundance comparison of sulfur metabolism genes between the marine ecosystem and non-marine ecosystem. The P values are based on Tukey–Kramer test. (**b**) Principal co-ordinates analysis (PCoA) of sulfur cycling genes. The R and P values in the figure are calculated based on ANOSIM. (**c**) Differences in the distribution of key sulfur genes in five habitats. The abundance is the average of each group. SE is expressed as error bars. UF, upland forest; DS, deep-sea sediments; MW, marine waters; RS, river sediments; MS, mangrove sediments.

SMDB was applied to profile sulfur cycle from five habitats: upland forest, deep-sea sediments, marine waters, river sediments, and mangrove sediments (Supplementary Table S4). The results show that the number of sulfur cycling genes were 110–159 in the five habitats. The top 10 sulfur genes were sulfonate transport system ATP-binding protein (*ssuB*)*,* Heterodisulfide reductase subunit A (*hdrA*)*,* Arylsulfatase (*atsA*)*,* 3-(methylthio)propionyl-CoA ligase (*dmdB*)*,* Adenylylsulfate kinase (*cysC*)*,* cysteine synthase (*cysE*)*,* Anaerobic dimethyl sulfoxide reductase subunit A (*dmsA*)*,* Sulfur-carrier protein (*tusA*)*,* S-sulfolactate dehydrogenase (*slcC*), and Heterodisulfide reductase iron-sulfur subunit D (*hdrD)*. The results showed that five of the top 10 genes belonged to organic degradation/synthesis pathway. Therefore, organic degradation/synthesis was the main sulfur metabolism conversion pathway in these five habitats. The sulfur cycling genes were significantly different (ANOSIM, permutations = 999, *p*-value = 0.001) among the five habitats (Fig. 5b). When grouped by habitats, the sulfur cycling genes were differentially enriched in different environments (Fig. 5). For example, the abundance of dissimilatory sulfate reduction genes (*dsrB*, *aprA/B* and *sat*) in the marine ecosystem (ME) were significantly higher than those of the non-marine ecosystem (NME) area. In comparison, the abundance of sulfur oxidation genes (*sqr*, *SOX,* and *soxC*) in the non-marine ecosystem were significantly higher than those of the marine ecosystem area (*p* < 0.05). Mangrove sediments exhibited the highest abundance of genes involved in dissimilatory sulfate reduction genes (*dsrA*, and *aprA/B*), with deep-sea sediments having a particularly high abundance of dissimilatory sulfate reduction gene (*dsrB*). River sediments exhibited the highest abundance of genes involved in sulfide oxidation (*soxB*), and DMSP conversion (*dmdB/C*).

#### Sulfur-metabolizing microorganism abundance and diversity

The composition of sulfur-metabolizing microorganisms showed that Proteobacteria was the dominant phylum of sulfur-metabolizing microorganism communities in the mangrove sediments, marine waters, and river sediments (Supplementary Fig. S9). Furthermore, chloroflexi was the dominant phylum of sulfur-metabolizing microorganism communities in deep-sea sediments.

**Figure 6:**
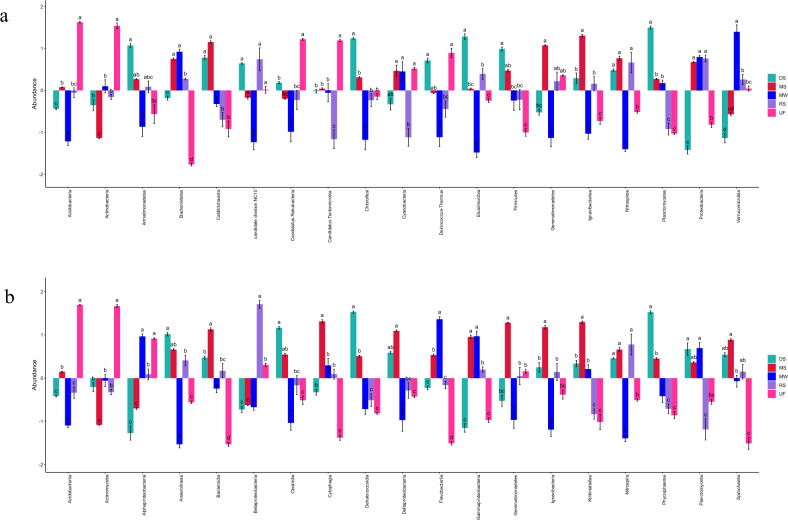
Differences in the distribution of sulfur metabolizing microorganisms in different habitats. The map is according to log^10^ of the 20 most abundant sulfur metabolizing microorganisms. SE is expressed as error bars. (**a**) Phylum level. (**b**) Class level. The color of the abundance bar represents habitats. Different lowercase black letters represent significant differences among habitats (*p* < 0.05). UF, upland forest; DS, deep-sea sediments; MW, marine waters; RS, river sediments; MS, mangrove sediments.

**Figure 7:**
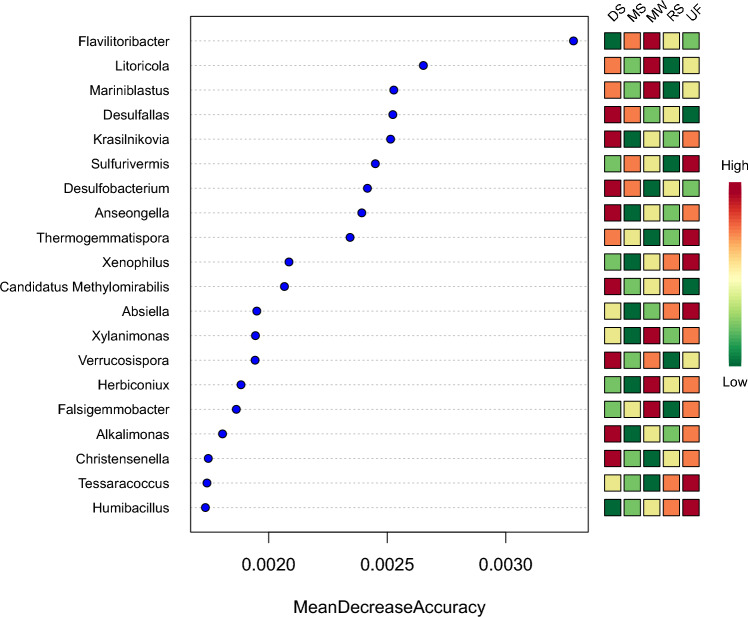
The features of sulfur metabolizing microorganisms are ranked by their frequencies of being selected as the random forest classifiers. The colored boxes on the right indicate the relative abundance ratio of the corresponding factor in each group.

At the phylum level, the abundance of Proteobacteria in mangrove sediments was significantly higher than in the deep-sea sediments and upland forest. The abundance of Nitrospirae in mangrove sediments was significantly higher than in the marine waters and upland forests. The abundance of Bacteroidetes in mangrove sediments was significantly higher than in the deep-sea sediments, river sediments, and upland forests. By contrast, the abundance of Actinobacteria in mangrove sediments was significantly lower than that of the marine waters, deep-sea sediments, river sediments, and upland forest (Fig. 6a). At the class level, the abundance of Gemmatimonadetes, Deltaproteobacteria, and Nitrospira in mangrove sediments were significantly higher than those of in marine waters. However, the abundance of Alphaproteobacteria and Betaproteobacteria in mangrove sediments were significantly lower than those in river sediments and upland forest (Fig. 6b). Random forest is a popular machine learning model that uses bootstrap aggregation and randomization of predictors to achieve a high degree of prediction accuracy^46^. The features that contribute the most to the sample grouping prediction's accuracy were selected using the random forest method. Notably, for the feature ranking method, the top factor -the *Flavilitoribacter* (phylum Bacteroidetes) contributed to the random forest models (Fig. 7). Figure 7 also shows that the addition of the signature of the microbial *Litoricola* and *Mariniblastus* into the models enabled us to achieve the highest accuracy. The results as mentioned above showed that SMDB was a powerful tool for analyzing sulfur cycle from metagenomic data in various environments.

## Discussion

Many microbes play an important role in the sulfur cycle^47,48^. Obtaining the complete list of sulfur cycle genes and sulfur-metabolizing microorganisms are critical to understanding sulfur cycle processes in the environment. In this study, a database was manually created for the fast and accurate analysis of sulfur cycle from metagenomic data. To our knowledge, SMDB online database contributes to the analysis of genomes and metagenomes, thereby screening for sulfur genes in large-scale sequence data sets.

The SMDB presents more comprehensive coverage of genes involved in sulfur metabolism than other public databases (Fig. 2). A new database, SCycDB^23^, containing 207 sulfur cycling genes, was constructed synchronously with the SMDB. We also found some comparable advantages when comparing with SCycDB (Fig. 2). For example, 32 sulfur genes (e.g., *SUOX, APA1_2, aprM, aps, ETHE1, MET10, MET3, MET5, npsr*) were not detected in SCycDB, such as the sulfite oxidase (*SUOX*) gene, which catalyzed the final step in cysteine catabolism, thereby oxidizing sulfite to sulfate^49^. The number of sulfur genes in the SCycDB database is higher than that in the SMDB database, mainly due to the addition of proteins involved in sulfur transfer, taurine, (R)-DHPS, choline-o-sulfate, PEP, and UDP-glucose metabolism. However, *tauD*, a gene that converts taurine and sulfite^50^, was also selected for the SMDB database. The SMDB database focuses on the key genes involved in converting sulfur compounds according to the references and sulfur metabolic pathways in KEGG and MetaCyc databases. We aim to provide the latest knowledge and research progress in sulfur metabolism studies, such as aerobic DMS degradation^51^. The product of DMS degradation (i.e., sulfuric and methanesulfonic acids) attracts water and promotes cloud formation, thereby affecting the climate. In addition, the sulfur sequences from the NR database have been selected to SMDB to facilitate sulfur-metabolizing microorganism annotation. An appropriate database is critical for the accuracy of metagenomic annotation. The SMDB primarily has the following three characteristics. Firstly, the SMDB has a precise definition of sulfur metabolism genes. Typical examples include *phsA* and *psrA* genes that encode the particle thiosulfate reductase and polysulfide reductase, respectively, but these genes share high sequence similarity^24^. Hence, distinguishing the activities of these two enzymes during genome annotation is difficult. The phylogenetic tree showed a clear separation of the *psrA* gene from the *phsA* gene (Supplementary Fig. S7). This result indicated the possibility of faulty ecological explanations. In SMDB, we have precisely defined these genes to avoid mis-annotations. Secondly, the SMDB considers the problem of false positives. This study addresses this problem by adding homologous sequences from multiple public databases. Thirdly, the small size (140 MB) of the SMDB reduces the computational cost required to obtain the sulfur metabolism genes. 2 G of metagenomic data consume 60 s of BLAST time. Therefore, the SMDB presents comparable advantages with regard to data quantity and quality.

Although we have collected as many sulfur genes as possible, some sulfur genes may still need to be noticed. A DATA SHARING interface (https://smdb.gxu.edu.cn/) was provided to submit sulfur genes with experimentally confirmed information in SMDB database. We plan to update the SMDB database continuously with novel sulfur cycle genes from literature and sequences submitted in SMDB web.

As the application of metagenomics in the environment increases, fast obtaining the functional profiles from metagenomics is important for researchers. The database described in this article, SMDB, unifies the most publicly available sulfur genes and provides a reliable annotation service to investigate sulfur cycle in different environments. SMDB gathered the comprehensive inorganic and organic sulfur transformation genes used to analyze sulfur cycle in five types of environments. Our results revealed that 110–159 of sulfur genes were detected in these environments. In the five habitats, the DMSP conversion in a high abundance, two of the top 10 genes belong to this process (Supplementary Table S4). This shows that DMSP is one of the Earth's most abundant organosulfur molecules^52^. In addition, a significant difference in the distribution of sulfur genes and sulfur-metabolizing microorganisms was found in these environments. For example, dissimilatory sulfate reduction genes were highly abundant in mangrove sediments and deep-sea sediments, probably because of the high concentration of sulfate in the sea^35^.

Microorganism alpha diversity was significantly higher in mangrove sediments than in the other habitats, PCoA showed that mangrove sediments also differed significantly from that of other habitats (Fig. 3). This finding is in agreement with previous results^53^. In addition, NCM showed that bacterial community structure in mangrove sediments was mainly driven by stochastic processes (R^2^ = 0.745, Nm = 371,591). Microbial dispersal was higher in the mangrove ecosystem than in others habitats. The high temperature and nutrient availability in mangrove sediments may explain the higher microorganism diversity. It could also be because mangroves are located in buffer zones that connect land and sea^54^. The river water discharges nutrients upstream into the mangrove sediments. In addition, it was observed that microbial composition showed distinctive patterns among different habitats. The microbial community in mangrove ecosystems was predominantly composed of members of Deltaproteobacteria.

Previous studies demonstrated that the microorganisms involved in the sulfur cycle primarily belong to Proteobacteria, Firmicutes and Actinobacteria of bacteria^55^. Sulfur-metabolizing microorganisms (e.g., Gemmatimonadetes, Deltaproteobacteria, Bacteroidetes, and Nitrospira) in mangrove sediments were found to be significantly more abundant than those in other habitats (Fig. 6). The metabolic diversity of Deltaproteobacteria can provide a competitive advantage for survival in fluctuating habitats^56^. Previous studies demonstrated that Deltaproteobacteria were associated with higher salinity^57^. Deltaproteobacteria are sulfate-reducing bacteria (SRB) with a potential for sulfate reduction, and organic matter decomposition^58^. The Bacteroidetes are considered primary degraders of polysaccharides and are found in many ecosystems^59^. Adding the signature of the microbial *Flavilitoribacter* into the models enabled the highest accuracy possible that it has a potential for polysaccharides.

Sulfur cycle is widely used in heavy metal contamination^16^. Given the increase of human activities, the sulfur cycle balance is affected, such as the lack of sulfur elements in the soil ecosystem leading to crop production reduction^60,61^. The SMDB will facilitate research to understand the sulfur cycle in different environments. Hence, this database will allow microbiologists to obtain the complete sulfur cycling genes comprehensively.

## Conclusion

A high sequences coverage of database, namely, SMDB, which focuses on the information of sulfur cycle, has been developed. This integrative database contains 175 genes and covers 11 sulfur metabolism processes. In addition, an online website database of SMDB was provided to analyze sulfur metabolism. The SMDB can analyze the sulfur metabolism quickly and accurately. Applying the SMDB to sulfur cycle in five diverse environments, it has demonstrated its ability to annotate sulfur cycle from metagenomes in different environments. SMDB will be a valuable resource for studying sulfur metabolisms from shotgun metagenomic data.

## Supplementary Information

Supplementary Information.

## Data Availability

Metagenomic data is available at NCBI, accession numbers: PRJEB24179, PRJNA485648, SRP068645, PRJEB41565, SRP190174, SRP190175, SRP190176, SRP190179, and SRP190180. Metagenomic data is available at Chinese National Genomics Data Center GSA database (https://bigd.big.ac.cn/gsub/), accession numbers: PRJCA002311. SMDB database is available at https://github.com/taylor19891213/sulfur-metabolism-gene-database and https://smdb.gxu.edu.cn/.

## Abbreviations

ORF: Open reading frame
SMDB: Sulfur metabolism gene integrative database
COG: Clusters of Orthologous Groups
eggNOG: Evolutionary Genealogy of Genes: Non-supervised Orthologous Groups
KEGG: Kyoto Encyclopedia of Genes and Genomes
NR: RefSeq non-redundant proteins database
UniProt: Universal protein database
