# Supplementary material for: Sulfur metabolism in subtropical marine mangrove sediments fundamentally differs from other habitats as revealed by SMDB
Source: Sci Rep. 2023 May 19;13:8126. doi: 10.1038/s41598-023-34995-y (PMC10199032; doi:10.1038/s41598-023-34995-y)
Supplement: Supplementary file 1 — Supplementary Information. [file 41598_2023_34995_MOESM1_ESM.pdf]

Supplementary Materials

Sulfur metabolism in subtropical marine mangrove sediments fundamentally differs from other habitats as revealed by SMDB

Shuming Mo [1, 2], Bing Yan[1, 3], Tingwei Gao [3], Jinhui Li [2], Muhammad Kashif [1, 2], Jingjing Song [6], Lirong Bai [6], Dahui Yu [6*], Jianping Liao [4, 5*], and Chengjian Jiang [1, 2, 6 **]

[1] Guangxi Research Center for Biological Science and Technology, Guangxi Academy of Sciences, 530007, China.

[2] State Key Laboratory for Conservation and Utilization of Subtropical Agro-bioresources, Guangxi Research Center for Microbial and Enzyme Engineering Technology, College of Life Science and Technology, Guangxi University, Nanning 530004, China.

[3] Guangxi Key Lab of Mangrove Conservation and Utilization, Guangxi Mangrove Research Center, Guangxi Academy of Sciences, Beihai 536000, China.

[4] Guangxi Key Lab of Human-machine Interaction and Intelligent Decision, Nanning Normal University, Nanning, 530299, China.

[5] State Key Laboratory of Radiation Medicine and Protection, Soochow University, Suzhou, 215123, China.

[6] Guangxi Key Laboratory of Beibu Gulf Marine Biodiversity Conservation, Beibu Gulf University, 535011, China.

*: Corresponding Author:

Tel: +86-771-3239403; Fax: +86-771-3237873

Email: jiangcj0520@vip.163.com (CJ**); ljp021916@163.com (JL*); pearlydh@163.com (DY*)

Supplementary Table S1. Summary of shotgun metagenome sequencing data used in this study.

Sample	Group	Project	SAR ID	Reads number	Location
UF1	Upland forest	PRJEB24179	ERR2233333	45329199	Hubbard Brook Experimental Forest, USA
UF2	Upland forest	PRJEB24179	ERR2233334	49284832	
UF3	Upland forest	PRJEB24179	ERR2233335	42439925	
UF4	Upland forest	PRJEB24179	ERR2233336	62330867	
UF5	Upland forest	PRJEB24179	ERR2233338	41467495	
DS1	Deep-sea sediments	PRJNA485648	SRR7716469	43341283	Eastern Gulf of Mexico
DS2	Deep-sea sediments	PRJNA485648	SRR7716470	74576455	
DS3	Deep-sea sediments	PRJNA485648	SRR7716471	69531635	
DS4	Deep-sea sediments	SRP068645	SRR12059190	28832090	Baltic Sea
DS5	Deep-sea sediments	SRP068645	SRR12059191	40486926	
MW1	Marine waters	PRJEB41565	ERR5008526	15533589	North Atlantic Ocean and Arctic Ocean
MW2	Marine waters	PRJEB41565	ERR5008524	11238651	
MW3	Marine waters	PRJEB41565	ERR5006650	18338688	
MW4	Marine waters	PRJEB41565	ERR5005971	19351837	
MW5	Marine waters	PRJEB41565	ERR5005153	21094890	
RS1	River sediments	SRP190174	SRR8837334	28718955	Ganga and Yamuna river, India
RS2	River sediments	SRP189880	SRR8815513	33887572	
RS3	River sediments	SRP191076	SRR8859357	24929338	
RS4	River sediments	SRP191079	SRR8859387	29128182	
RS5	River sediments	SRP191075	SRR8859356	54469302	

MS1	Mangrove sediments	PRJCA002311	SAMC187053	39584542	National Shankou Natural Reserve of Mangrove, China
MS2	Mangrove sediments	PRJCA002311	SAMC187054	67058935	
MS3	Mangrove sediments	PRJCA002311	SAMC187055	60558772	
MS4	Mangrove sediments	PRJCA002311	SAMC187056	37337451	
MS5	Mangrove sediments	PRJCA002311	SAMC187057	70467923	

Supplementary Table S2. Summary of sulfur cycling genes in SMDB and other databases.

Pathway	Gene	Annotation	Core database sequences	Full database sequences	Other databases				
					NR	M5nr	KEGG	eggNOG	COG
**Assimilatory sulfate reduction**	APA1_2	Sulfate adenylyltransferase (ADP) / ATP adenylyltransferase	3	9	6	0	0	0	0
	APR	Adenylyl-sulfate reductase (glutathione)	9	152	69	36	0	38	0
	cysC	Adenylylsulfate kinase	16251	38287	15562	3098	1433	1636	307
	cysD	Sulfate adenylyltransferase subunit 2	10249	18083	5633	1045	438	621	97
	cysH	Phosphoadenosine phosphosulfate reductase	7392	27424	14130	2929	1462	1312	199
	cysI	Sulfite reductase (NADPH) hemoprotein beta-component	4716	18377	9782	2031	945	780	123
	cysJ	Sulfite reductase (NADPH) flavoprotein alpha-component	3530	23717	15032	3045	1285	704	121
	cysN	Sulfate adenylyltransferase subunit 1	9800	21823	8823	1576	642	855	127
	cysNC	Bifunctional enzyme CysN/CysC	1034	2571	1286	111	47	85	8
	HINT4	Sulfate adenylyltransferase (ADP) / adenylylsulfatase	1	18	9	3	0	5	0
	MET10	Sulfite reductase [NADPH] flavoprotein component	16	146	93	24	5	8	0
	MET3	Sulfate adenylyltransferase	852	1061	118	34	16	40	1

	MET5	Sulfite reductase subunit beta	17	128	80	21	2	8	0
	PAPSS	3'-phosphoadenosine 5'-phosphosulfate synthase	246	1847	1055	349	0	197	0
	sat	Sulfate adenylyltransferase	20431	51745	21785	4434	2118	2606	371
	nrnA	Bifunctional oligoribonuclease and PAP phosphatase NrnA	4431	18124	9477	2240	964	895	117
	cysQ	3'(2'), 5'-bisphosphate nucleotidase	25425	51246	18616	3460	1645	1826	274
	IMPAD1	Inositol monophosphatase 3	86	563	277	114	0	86	0
	SAL	3'(2'), 5'-bisphosphate nucleotidase / inositol polyphosphate 1-phosphatase	7	101	47	17	0	30	0
	MET22	3'(2'), 5'-bisphosphate nucleotidase	75	290	145	38	11	21	0
	BPNT1	3'(2'), 5'-bisphosphate nucleotidase	165	925	503	168	0	89	0
	sir	Sulfite reductase (ferredoxin)	1309	13463	9048	1649	631	758	68
	sirA	Sulfite reductase (ferredoxin)	56	513	282	86	39	45	5
	aps	ATP sulfurylase	35	371	172	80	0	84	0
**Dissimilatory sulfate reduction**	aprA	Adenylylsulfate reductase, subunit A	151	3769	2483	1041	30	50	14
	aprB	Adenylylsulfate reductase, subunit B	53	959	517	182	81	104	22
	aprM	Adenylylsulfate reductase membrane anchor	4	33	10	8	3	8	0
	dsrA	Dissimilatory sulfite reductase alpha subunit	5278	16480	6390	4589	73	109	41
	dsrB	Dissimilatory sulfite reductase beta subunit	6696	19954	8582	4458	76	108	34
	dsrC	Dissimilatory sulfite reductase subunit c	4	9	3	2	0	0	0

	dsrD	Dissimilatory sulfite reductase subunit D (DsrD)	1	3	1	1	0	0	0
	dsrJ	Sulfite reduction-associated complex DsrMKJOP multiheme protein DsrJ	41	115	39	17	7	11	0
	dsrK	Sulfite reduction-associated complex DsrMKJOP multiheme protein DsrK	75	341	142	51	19	43	11
	dsrM	Sulfite reduction-associated complex DsrMKJOP protein DsrM	68	187	75	23	5	14	2
	dsrN	Dissimilatory sulfite reductase subunit N	1	6	1	1	1	1	1
	dsrO	Sulfite reduction-associated complex DsrMKJOP iron-sulfur protein DsrO	63	133	47	11	3	8	1
	dsrP	Sulfite reduction-associated complex DsrMKJOP protein DsrP	71	205	86	21	8	15	4
	qmoA	Adenylylsulfate reductase-associated electron transfer protein QmoA	1	7	3	1	1	1	0
	sat	Sulfate adenylyltransferase	20431	51745	21785	4434	2118	2606	371
**Organic degradation/synthesis**	ATCYSC1	L-3-cyanoalanine synthase/ cysteine synthase	4	58	29	10	0	15	0
	atsA	Arylsulfatase	2660	21439	14005	3094	817	768	95
	atsK	Alpha-ketoglutarate-dependent sulfate ester dioxygenase	23	366	271	45	5	22	0
	comC	R-sulfolactate oxidoreductase, comC	4	31	9	6	6	4	2
	comD	Sulfopyruvate decarboxylase	180	675	317	78	36	52	12

	subunit alpha							
comE	Sulfopyruvate decarboxylase subunit beta	163	519	231	47	31	35	12
cuyA	L-cysteate sulfo-lyase	1	39	23	4	2	7	2
cysE	cysteine synthase	11057	42608	20639	5322	2740	2492	358
cysK	Cysteine synthase	1462	27195	17066	4219	2388	1781	279
dddD	Dimethylsulfoniopropanoate:acyl-CoA transferase, dddD	1	6	1	1	1	1	0
dddL	Dimethylpropiothetin dethiomethylase	159	344	156	15	3	11	1
dddP	Dimethylsulfoniopropionate lyase	73	677	511	45	7	38	3
dddQ	Dimethylsulfoniopropionate lyase	18	107	75	6	2	5	1
dddW	Dimethlysulfonioproprionate lyase DddW	20	75	40	7	3	4	1
dddY	Dimethylsulfoniopropionate lyase	5	31	22	2	0	2	0
ddhA	Dimethylsulfide dehydrogenase subunit alpha	29	220	149	25	7	8	2
ddhB	Dimethylsulfide dehydrogenase subunit beta	6	49	22	14	3	3	1
ddhC	Dimethylsulfide dehydrogenase subunit gamma	5	21	11	2	1	2	0
dmdA	Dimethylsulfoniopropionate demethylase	511	1122	487	71	9	39	5
dmdB	3-(methylthio)propionyl---CoA ligase	91	2799	2179	299	113	112	5
dmdC	3-(methylthio)propanoyl-CoA dehydrogenase	1	6	1	1	1	1	1
dmdD	(Methylthio)acryloyl-CoA hydratase	1	10	4	2	1	1	1

dmoA	Dimethyl-sulfide monooxygenase	3	540	461	53	6	17	0
dmoB	Dissimilatory dimethyl-sulfide monooxygenase	1	4	2	1	0	0	0
dmsA	Anaerobic dimethyl sulfoxide reductase subunit A	1374	24534	17993	3848	873	358	88
dmsB	Anaerobic dimethyl sulfoxide reductase subunit B	3	3002	2293	368	266	60	12
dmsC	Anaerobic dimethyl sulfoxide reductase subunit C	796	9945	6922	1470	576	147	34
dsoB	DMS oxygenase β subunit	1	11	6	3	0	1	0
dsoC	DMS oxygenase γ subunit	1	42	28	8	0	5	0
dsoD	DMS oxygenase component	1	303	209	75	4	14	0
dsoE	DMS oxygenase ε subunit	1	11	6	3	0	1	0
dsoF	DMSO monooxygenase reductase component	1	17	9	5	0	2	0
mccB	Cystathionine gamma-lyase / homocysteine desulfhydrase	96	1833	1198	257	183	86	13
mddA	Methanethiol S-methyltransferase	6	152	83	16	40	6	1
MET17	O-acetylhomoserine/O-acetylserine sulfhydrylase	3	104	54	44	3	0	0
msmA	Methanesulfonate monooxygenase subunit alpha	33	148	84	13	7	11	0
msmB	Methanesulfonate monooxygenase subunit beta	6	19	8	2	1	1	1
msuD	Methanesulfonate monooxygenase	292	3118	2192	324	124	180	6
mtsA	Methylthiol:coenzyme M methyltransferase	29	78	37	5	4	2	1
mtsB	Methylated-thiol--corrinoid protein	2	13	5	3	2	1	0

	SELENBP1	Methanethiol oxidase	33	429	223	108	0	65	0
	sfnG	Dimethylsulfone monooxygenase	19	544	405	73	22	23	2
	slcC	S-sulfolactate dehydrogenase	220	1441	973	143	42	54	9
	slcD	sulfolactate dehydrogenase	2	17	3	4	3	3	2
	cysO	Cysteine synthase / O-phosphoserine sulfhydrylase / cystathionine beta-synthase	1	8	3	1	1	1	1
	ssuA	Sulfonate transport system substrate-binding protein	131	7832	5984	977	539	159	42
	ssuB	Sulfonate transport system ATP-binding protein	127	5631	4115	736	430	193	30
	ssuC	Sulfonate transport system permease protein	1552	20416	13582	2942	1445	792	103
	ssuD	Alkanesulfonate monooxygenase	12583	62941	39079	5765	2266	2976	272
	ssuE	FMN reductase	1791	9770	5639	1159	644	484	53
	suyA	(2R)-sulfolactate sulfo-lyase subunit alpha	82	388	204	40	20	35	7
	suyB	(2R)-sulfolactate sulfo-lyase subunit beta	141	1199	749	155	51	88	15
	tauD	Taurine dioxygenase	7011	36949	21645	4311	1992	1798	192
	Tmm	Trimethylamine monooxygenase	24	81	29	21	3	3	1
	tmoC	Toluene monooxygenase system ferredoxin subunit	30	98	48	10	2	7	1
	tmoF	Toluene monooxygenase electron transfer component	2	6	3	1	0	0	0
	xsc	Sulfoacetaldehyde acetyltransferase	1053	4261	2423	388	128	233	36
**Sulfide oxidation**	ETHE1	Sulfur dioxygenase	75	511	254	99	4	78	1
	fccA	Cytochrome subunit of sulfide	86	342	176	46	13	15	6

		dehydrogenase							
	fccB	Sulfide dehydrogenase [flavocytochrome c] flavoprotein chain	502	5276	3638	597	318	185	36
	sdo	Sulfur dioxygenase	1	9	4	2	1	1	0
	SOX	Sulfite oxidase Sox	20	156	81	24	3	27	1
	SQOR	Eukaryotic sulfide quinone oxidoreductase	18	361	178	101	1	63	0
	sqr	Sulfide:quinone oxidoreductase	195	1341	710	229	97	81	29
	SUOX	Sulfite oxidase	118	975	573	168	15	100	1
	TST	Thiosulfate/3-mercaptopyruvate sulfurtransferase	1	19	14	1	1	1	1
**Sulfite oxidation**	aprA	Adenylylsulfate reductase, subunit A	151	3769	2483	1041	30	50	14
	aprB	Adenylylsulfate reductase, subunit B	53	959	517	182	81	104	22
	aprM	Adenylylsulfate reductase membrane anchor	4	33	10	8	3	8	0
	apt	Adenylylsulfate:phosphate adenylyltransferase	1	6	4	1	0	0	0
	sat	Sulfate adenylyltransferase	20431	51745	21785	4434	2118	2606	371
	SoeA	Sulfite dehydrogenase (quinone) subunit SoeA	1	19	5	5	2	4	2
	SoeB	Sulfite dehydrogenase (quinone) subunit SoeB	3	42	21	6	3	6	3
	soeC	Sulfite dehydrogenase (quinone) subunit SoeC	2	12	3	2	2	2	1
	sorA	Sulfite dehydrogenase	289	1605	973	134	67	113	29

	sorB	Sulfite dehydrogenase	200	934	533	92	39	62	8
	sorT	Sulfite dehydrogenase	2	63	26	25	1	9	0
	SOX	Sulfite oxidase Sox	20	156	81	24	3	27	1
	SUOX	Sulfite oxidase	118	975	573	168	15	100	1
**Sulfur oxidation**	dsrA	Dissimilatory sulfite reductase alpha subunit	5278	16480	6390	4589	73	109	41
	dsrB	Dissimilatory sulfite reductase beta subunit	6696	19954	8582	4458	76	108	34
	dsrC	Dissimilatory sulfite reductase subunit c	4	9	3	2	0	0	0
	dsrE	Sulfurtransferase	1	40	17	8	4	7	3
	dsrF	Sulfurtransferase	603	2762	1567	302	142	126	22
	dsrH	Sulfurtransferase	1	7	2	1	1	1	1
	dsrJ	Sulfite reduction-associated complex DsrMKJOP multiheme protein DsrJ	41	115	39	17	7	11	0
	dsrK	Sulfite reduction-associated complex DsrMKJOP multiheme protein DsrK	75	341	142	51	19	43	11
	dsrM	Sulfite reduction-associated complex DsrMKJOP protein DsrM	68	187	75	23	5	14	2
	dsrO	Sulfite reduction-associated complex DsrMKJOP iron-sulfur protein DsrO	63	133	47	11	3	8	1
	dsrP	Sulfite reduction-associated complex DsrMKJOP protein DsrP	71	205	86	21	8	15	4
	ETHE1	Sulfur dioxygenase	75	511	254	99	4	78	1
	hdrA	Heterodisulfide reductase subunit A	335	1480	641	216	134	102	52

	hdrB	Heterodisulfide reductase subunit B	4	163	61	53	7	36	2
	hdrC	Heterodisulfide reductase subunit C	293	1127	453	129	125	84	43
	hdrD	Heterodisulfide reductase iron-sulfur subunit D	142	509	235	52	39	22	19
	hdrE	Heterodisulfide reductase subunit E	23	106	51	12	9	5	6
	hdrF	Heterodisulfide reductase, cytochrome reductase subunit	1	4	3	0	0	0	0
	npsr	NADH-dependent persulfide reductase [flavoprotein/rhodanase]	1	15	10	1	1	2	0
	sqr	Sulfide:quinone oxidoreductase	195	1341	710	229	97	81	29
	rhd	Rhodanese	2	29	15	5	3	3	1
	sdo	Sulfur dioxygenase	1	9	4	2	1	1	0
	sgpA	Sulfur globule protein	1	5	1	1	1	1	0
	sgpB	Sulfur globule protein	1	1	0	0	0	0	0
	sgpC	Sulfur globule protein	1	1	0	0	0	0	0
	sor	Sulfur oxygenase/reductase	15	74	26	21	5	6	1
	tusA	Sulfur-carrier protein	11711	30759	12360	2816	1967	1655	250
**Sulfur reduction**	hydA	Sulfhydrogenase subunit alpha	7	37	18	3	4	3	2
	hydB	Sulfhydrogenase subunit beta (sulfur reductase)	9	68	31	8	10	8	2
	hydD	Sulfhydrogenase subunit delta	8	59	27	7	8	6	3
	hydG	Sulfhydrogenase subunit gamma (sulfur reductase)	26	201	92	27	27	21	8
	psrA	Polysulfide reductase chain A	11	279	208	53	1	6	0
	psrB	Polysulfide reductase chain B	4	33	10	6	6	4	3
	psrC	Polysulfide reductase chain C	3	28	11	5	4	3	2
	shyB	Sulfhydrogenase II β subunit	12	28	12	1	2	1	0

	shyC	Sulfhydrogenase II γ subunit	35	81	38	2	3	1	2
	sor	Sulfur oxygenase/reductase	15	74	26	21	5	6	1
	sreA	Sulfur reductase molybdopterin subunit	7	57	20	9	8	8	5
	sreB	Sulfur reductase FeS subunit	1	4	2	1	0	0	0
	sreC	Sulfur reductase membrane anchor	1	4	2	1	0	0	0
**Tetrathionate oxidation**	doxA	Thiosulfate dehydrogenase [quinone] small subunit	2	9	3	2	1	1	0
	doxD	Thiosulfate dehydrogenase [quinone] large subunit	12	385	227	72	65	9	0
	dsrE	Sulfurtransferase	1	40	17	8	4	7	3
	tetH	Tetrathionate hydrolase	5	31	12	7	3	3	1
	tsdA	Thiosulfate dehydrogenase	29	547	384	67	41	20	6
	TST	Thiosulfate/3-mercaptopyruvate sulfurtransferase	1	19	14	1	1	1	1
	tusA	Sulfur-carrier protein	11711	30759	12360	2816	1967	1655	250
	glpE	Thiosulfate sulfurtransferase	9311	27591	12065	2945	1895	1221	154
**Tetrathionate reduction**	asrA	Anaerobic sulfite reductase subunit A	798	2979	1499	423	125	126	8
	asrB	Anaerobic sulfite reductase subunit B	799	2849	1359	378	138	157	18
	asrC	Anaerobic sulfite reductase subunit C	415	2234	1175	363	146	117	18
	glpE	Thiosulfate sulfurtransferase	9311	27591	12065	2945	1895	1221	154
	otr	Octaheme tetrathionate reductase	3	58	33	11	8	3	0
	phsA	Thiosulfate reductase / polysulfide reductase chain A	4	8	4	0	0	0	0
	phsB	Thiosulfate reductase electron	623	2236	1021	343	153	72	24

		transport protein						
	phsC	Thiosulfate reductase cytochrome b subunit	67	1377	916	235	133	20	6
	TST	Thiosulfate/3-mercaptopyruvate sulfurtransferase	1	19	14	1	1	1	1
	ttrA	Tetrathionate reductase subunit A	1460	5477	3132	584	150	131	20
	ttrB	Tetrathionate reductase subunit B	611	3361	2054	377	147	140	32
	ttrC	Tetrathionate reductase subunit C	462	2445	1545	287	99	45	7
**Thiosulfate disproportionation**	glpE	Thiosulfate sulfurtransferase	9311	27591	12065	2945	1895	1221	154
	MPST	3-mercaptopyruvate sulfurtransferase	3	3	0	0	0	0	0
	phsA	Thiosulfate reductase / polysulfide reductase chain A	4	8	4	0	0	0	0
	phsB	Thiosulfate reductase electron transport protein	623	2236	1021	343	153	72	24
	phsC	Thiosulfate reductase cytochrome b subunit	67	1377	916	235	133	20	6
	pspE	Thiosulfate sulfurtransferase	1121	6429	3573	886	490	320	39
	RDL	Sulfurtransferase	6	42	22	10	2	2	0
	sseA	Thiosulfate/3-mercaptopyruvate sulfurtransferase	477	9732	6382	1526	791	471	85
	ygaP	Thiosulfate sulfurtransferase YgaP	1	549	362	94	78	11	0
**Thiosulfate oxidation**	doxA	Thiosulfate dehydrogenase [quinone] small subunit	2	9	3	2	1	1	0
	doxD	Thiosulfate dehydrogenase [quinone] large subunit	12	385	227	72	65	9	0
	glpE	Thiosulfate sulfurtransferase	9311	27591	12065	2945	1895	1221	154
	MPST	3-mercaptopyruvate	3	3	0	0	0	0	0

	sulfurtransferase							
phsA	Thiosulfate reductase / polysulfide reductase chain A	4	8	4	0	0	0	0
phsB	Thiosulfate reductase electron transport protein	623	2236	1021	343	153	72	24
phsC	Thiosulfate reductase cytochrome b subunit	67	1377	916	235	133	20	6
rdlA	Rhodanese	7	29	15	7	0	0	0
soxA	L-cysteine S-thiosulfotransferase	789	2632	1358	215	89	154	27
soxB	Thiosulfohydrolase	224	1848	1021	322	110	128	43
soxD	Sulfur dehydrogenase subunit SoxD	49	401	179	72	43	42	16
soxS	Sulfur/thiosulfate oxidation protein SoxS	2	6	2	2	0	0	0
soxV	Sulfur/thiosulfate oxidation protein SoxV	2	6	2	2	0	0	0
soxW	Sulfur/thiosulfate oxidation protein SoxW	2	6	2	2	0	0	0
soxX	Sulfur oxidation protein SoxX	313	1922	860	314	177	200	58
soxY	Sulfur-oxidizing protein SoxY	664	2984	1861	230	65	152	12
tsdA	Thiosulfate dehydrogenase	29	547	384	67	41	20	6
tsdB	Thiosulfate dehydrogenase electron acceptor	9	127	76	18	10	11	3
TST	Thiosulfate/3-mercaptopyruvate sulfurtransferase	1	19	14	1	1	1	1
SoxC	Sulfane dehydrogenase subunit SoxC	206	1256	816	105	43	74	12
soxL	Sulfur transferase, periplasm	1	6	1	1	1	1	1

Supplementary Table S3. Taxonomic composition of sulfur cycling pathways in SMDB.

Pathway		Phylum	Class	Order	Family	Genus
		Euryarchaeota	Halobacteria	Nitrosopumilales	Nitrosopumilaceae	Nitrosopumilus
		Thaumarchaeota	Thermoprotei	Haloferacales	Haloferacaceae	Haloferax
		Crenarchaeota	Thermococci	Thermococcales	Thermococcaceae	Thermococcus
**Assimilatory sulfate reduction**	Archaea	Candidatus Bathyarchaeota	Candidatus Poseidoniia	Candidatus Poseidoniales	Sulfolobaceae	Haloarcula
		Candidatus Altiarchaeota	Methanomicrobia	Methanosarcinales	Haloarculaceae	Sulfolobus
		Candidatus Thorarchaeota	Thermoplasmata	Halobacteriales	Nitrososphaeraceae	Methanococcus
		Candidatus	Methanococci	Desulfurococcal	Methanosarcinac	Nitrosarchaeum

	Woesearchaeota		es	eae	
	Candidatus Heimdallarchaeota	Nitrososphaeria	Methanococcales	Methanococcaceae	Acidilobus
	Candidatus Lokiarchaeota	Archaeoglobi	Sulfolobales	Candidatus Thalassarchaeaceae	Candidatus Thalassarchaeum
	Candidatus Odinarchaeota	Methanobacteria	Acidilobales	Archaeoglobaceae	Methanothrix
				Acidilobaceae	Methanocaldococcus
	Proteobacteria	Gammaproteobacteria	Enterobacterales	Enterobacteriaceae	Pseudomonas
Bacteria	Actinobacteria <actinobacteria>	Actinobacteria	Pseudomonadales	Pseudomonadaceae	Escherichia
	Firmicutes	Alphaproteob	Bacillales	Bacillaceae	Bacillus

			acteria			
		Bacteroidetes	Bacilli	Burkholderiales	Vibrionaceae	Streptomyces
		Cyanobacteria	Betaproteobacteria	Rhizobiales	Burkholderiaceae	Vibrio
		Planctomycetes	Flavobacteriia	Corynebacteriales	Streptomycetaceae	Burkholderia
		Verrucomicrobia	Clostridia	Vibrionales	Mycobacteriaceae	Salmonella
		Spirochaetes	Epsilonproteobacteria	Streptomycetales	Rhizobiaceae	Staphylococcus
		Acidobacteria	Deltaproteobacteria	Alteromonadales	Moraxellaceae	Acinetobacter
		Chloroflexi	Bacteroidia	Xanthomonadales	Xanthomonadaceae	Klebsiella
**Dissimilatory**	Archaea	Euryarchaeota	Halobacteria	Archaeoglobale	Archaeoglobacea	Archaeoglobus

**sulfate reduction**			s	e	
	Thaumarchaeota	Archaeoglobi	Haloferacales	Nitrosopumilaceae	Nitrosopumilus
	Crenarchaeota	Methanomicrobia	Nitrosopumilales	Haloferacaceae	Thermococcus
	Candidatus Bathyarchaeota	Thermoprotei	Halobacteriales	Halorubraceae	Haloferax
	Candidatus Woesearchaeota	Candidatus Poseidoniia	Candidatus Poseidoniales	Thermococcaceae	Halorubrum
	Candidatus Altiarchaeota	Thermococci	Methanosarcinales	Haloarculaceae	Haloarcula
	Candidatus Lokiarchaeota	Thermoplasmata	Thermococcales	Natrialbaceae	Natrinema
	Candidatus Heimdallarchaeota	Methanococci	Natrialbales	Methanosarcinaceae	Methanosarcina

	Candidatus Thorarchaeota	Nitrososphaeria	Thermoproteales	Thermoproteaceae	Nitrosarchaeum
	Candidatus Verstraetearchaeota	Methanobacteria	Desulfurococcales	Methanotrichaceae	Methanothrix
		Nanohaloarchaea			Methanohalophilus
	Proteobacteria	Gammaproteobacteria	Enterobacterales	Enterobacteriaceae	Pseudomonas
	Firmicutes	Alphaproteobacteria	Rhizobiales	Pseudomonadaceae	Streptomyces
Bacteria	Actinobacteria <actinobacteria>	Actinobacteria	Pseudomonadales	Mycobacteriaceae	Bacillus
	Bacteroidetes	Bacilli	Bacillales	Bacillaceae	Vibrio
	Cyanobacteria	Betaproteobacteria	Corynebacteriales	Streptomycetaceae	Mycobacterium

		Planctomycetes	Clostridia	Burkholderiales	Vibrionaceae	Burkholderia
		Verrucomicrobia	Deltaproteobacteria	Clostridiales	Burkholderiaceae	Escherichia
		Spirochaetes	Flavobacteriia	Streptomycetales	Rhizobiaceae	Salmonella
		Acidobacteria	Epsilonproteobacteria	Vibrionales	Rhodobacteraceae	Campylobacter
		Tenericutes	Bacteroidia	Rhodobacterales	Flavobacteriaceae	Staphylococcus
**Organic degradation and synthesis**	Archaea	Euryarchaeota	Halobacteria	Halobacteriales	Haloferacaceae	Haloferax
		Thaumarchaeota	Methanomicrobia	Haloferacales	Haloarculaceae	Haloarcula
		Candidatus Lokiarchaeota	Methanobacteria	Methanosarcinales	Methanobacteriaceae	Methanosarcina
		Crenarchaeota	Methanococci	Methanobacteri	Methanosarcinac	Methanobrevibacte

			ales	eae	r
	Candidatus Heimdallarchaeota	Thermoplasmata	Natrialbales	Natrialbaceae	Methanobacterium
	Candidatus Thorarchaeota	Thermococci	Methanococcales	Halobacteriaceae	Natronorubrum
	Candidatus Odinarchaeota	Candidatus Methanofastidiosa	Thermococcales	Methanocaldococcaceae	Natrialba
		Nitrososphaeria	Methanomicrobiales	Methanotrichaceae	Methanothermobacter
		Candidatus Poseidoniia	Nitrosopumilales	Methanococcaceae	Methanocaldococcus
		Methanopyri	Methanomassiliicoccales	Thermococcaceae	Methanothrix
					Methanococcus

Bacteria	Proteobacteria	Gammaproteobacteria	Enterobacterales	Enterobacteriaceae	Pseudomonas
	Actinobacteria <actinobacteria>	Actinobacteria	Pseudomonadales	Pseudomonadaceae	Burkholderia
	Firmicutes	Betaproteobacteria	Burkholderiales	Burkholderiaceae	Escherichia
	Bacteroidetes	Alphaproteobacteria	Corynebacteriales	Streptomycetaceae	Streptomyces
	Cyanobacteria	Bacilli	Rhizobiales	Mycobacteriaceae	Klebsiella
	Spirochaetes	Clostridia	Bacillales	Bacillaceae	Salmonella
	Planctomycetes	Bacteroidia	Streptomycetales	Rhizobiaceae	Bacillus
	Verrucomicrobia	Epsilonproteobacteria	Micrococcales	Moraxellaceae	Acinetobacter

		Chloroflexi	Flavobacteriia	Vibrionales	Vibrionaceae	Mycobacterium
		Nitrospirae	Spirochaetia	Rhodobacterales	Rhodobacteraceae	Enterobacter
**Sulfide oxidation**	Archaea	Euryarchaeota	Halobacteria	Haloferacales	Haloferacaceae	Haloferax
		Crenarchaeota	Thermoprotei	Sulfolobales	Sulfolobaceae	Sulfolobus
		Candidatus Heimdallarchaeota	Thermoplasmata	Thermoplasmatales	Ferroplasmaceae	Acidianus
				Halobacteriales	Halobacteriaceae	Ferroplasma
						Halalkaliarchaeum
						Halodesulfurarchaeum
	Bacteria	Proteobacteria	Gammaproteobacteria	Pseudomonadales	Pseudomonadaceae	Pseudomonas
		Firmicutes	Alphaproteob	Bacillales	Bacillaceae	Bacillus

	acteria			
Actinobacteria <actinobacteria>	Bacilli	Burkholderiales	Moraxellaceae	Acinetobacter
Deinococcus-Thermus	Betaproteobacteria	Rhizobiales	Burkholderiaceae	Rhizobium
Cyanobacteria	Actinobacteria	Corynebacteriales	Mycobacteriaceae	Serratia
Bacteroidetes	Acidithiobacillia	Enterobacterales	Rhizobiaceae	Mycobacterium
Aquificae	Epsilonproteobacteria	Rhodobacterales	Yersiniaceae	Staphylococcus
Nitrospirae	Deinococci	Acidithiobacillales	Rhodobacteraceae	Mesorhizobium
Planctomycetes	Flavobacteriia	Campylobacterales	Staphylococcaceae	Cupriavidus

		Chlorobi	Aquificae	Thermales	Phyllobacteriaceae	Acidithiobacillus
		Euryarchaeota	Archaeoglobi	Archaeoglobales	Archaeoglobaceae	Archaeoglobus
		Thaumarchaeota	Thermoprotei	Nitrosopumilales	Nitrosopumilaceae	Thermococcus
		Crenarchaeota	Thermococci	Thermococcales	Thermococcaceae	Nitrosopumilus
**Sulfite oxidation**	Archaea	Candidatus Bathyarchaeota	Thermoplasmata	Sulfolobales	Sulfolobaceae	Sulfolobus
		Candidatus Heimdallarchaeota	Halobacteria	Desulfurococcales	Natrialbaceae	Nitrosarchaeum
		Candidatus Lokiarchaeota	Methanomicrobia	Acidilobales	Desulfurococcaceae	Acidilobus
		Candidatus	Candidatus	Natrialbales	Nitrososphaerace	Haloterrigena

	Thorarchaeota	Poseidoniia		ae	
	Candidatus Korarchaeota	Nitrososphaeria	Methanosarcinales	Acidilobaceae	Candidatus Nitrosotalea
	Nanoarchaeota	Methanococci	Thermoplasmatales	Pyrodictiaceae	Nitrososphaera
	Candidatus Verstraetearchaeota		Nitrososphaerales	Methanocaldococcaceae	Methanocaldococcus
	Proteobacteria	Gammaproteobacteria	Enterobacterales	Enterobacteriaceae	Pseudomonas
	Firmicutes	Alphaproteobacteria	Rhizobiales	Pseudomonadaceae	Bacillus
Bacteria	Actinobacteria <actinobacteria>	Bacilli	Bacillales	Bacillaceae	Vibrio
	Bacteroidetes	Actinobacteria	Pseudomonadales	Rhizobiaceae	Escherichia

		Cyanobacteria	Betaproteobacteria	Vibrionales	Vibrionaceae	Streptomyces
		Planctomycetes	Deltaproteobacteria	Alteromonadales	Moraxellaceae	Rhizobium
		Acidobacteria	Epsilonproteobacteria	Corynebacteriales	Streptomycetaceae	Acinetobacter
		Nitrospirae	Bacteroidia	Streptomycetales	Mycobacteriaceae	Mycobacterium
		Deinococcus-Thermus	Clostridia	Oceanospirillales	Paenibacillaceae	Salmonella
		Chloroflexi	Flavobacteriia	Burkholderiales	Bradyrhizobiaceae	Paenibacillus
**Sulfur oxidation**	Archaea	Euryarchaeota	Methanomicrobia	Methanosarcinales	Methanosarcinaceae	Methanosarcina
		Crenarchaeota	Methanobacte	Archaeoglobale	Methanobacteria	Archaeoglobus

	ria	s	ceae	
Candidatus Thorarchaeota	Archaeoglobi	Methanobacteriales	Archaeoglobaceae	Methanobrevibacter
Candidatus Lokiarchaeota	Thermoprotei	Methanomicrobiales	Sulfolobaceae	Methanothrix
Candidatus Bathyarchaeota	Methanococci	Sulfolobales	Methanotrichaceae	Methanobacterium
Candidatus Heimdallarchaeota	Thermoplasmata	Methanococcales	Methanoregulaceae	Acidianus
Candidatus Altiarchaeota	Halobacteria	Methanomassiliicoccales	Methanomicrobiaceae	Methanoregula
Candidatus Korarchaeota	Methanopyri	Halobacteriales	Thermoproteaceae	Methanoculleus
Candidatus Hydrothermarchaeot	Candidatus Methanofastid	Thermoproteales	Methanococcaceae	Sulfolobus

	a	iosa			
	Candidatus Aenigmarchaeota		Methanocellales	Haloarculaceae	Methanococcus
	Candidatus Verstraetearchaeota			Methanocellaceae	
	Candidatus Odinarchaeota				
Bacteria	Proteobacteria	Gammaproteobacteria	Pseudomonadales	Pseudomonadaceae	Pseudomonas
	Firmicutes	Deltaproteobacteria	Enterobacterales	Enterobacteriaceae	Bacillus
	Actinobacteria <actinobacteria>	Bacilli	Bacillales	Bacillaceae	Desulfovibrio
	Bacteroidetes	Alphaproteobacteria	Desulfobacterales	Desulfovibrionaceae	Escherichia

		Nitrospirae	Betaproteobacteria	Desulfovibrionales	Desulfobulbaceae	Vibrio
		Cyanobacteria	Clostridia	Clostridiales	Vibrionaceae	Desulfobulbus
		Aquificae	Actinobacteria	Burkholderiales	Moraxellaceae	Acinetobacter
		Chloroflexi	Acidithiobacillia	Alteromonadales	Desulfobacteraceae	Klebsiella
		Acidobacteria	Nitrospira	Vibrionales	Pasteurellaceae	Enterobacter
		Chlorobi	Aquificae	Rhizobiales	Peptococcaceae	Haemophilus
**Sulfur reduction**	Archaea	Euryarchaeota	Thermococci	Thermococcales	Thermococcaceae	Thermococcus
		Thaumarchaeota	Methanomicrobia	Nitrosopumilales	Nitrosopumilaceae	Nitrosopumilus
		Crenarchaeota	Thermoprotei	Methanosarcinales	Sulfolobaceae	Acidianus
		Candidatus	Candidatus	Sulfolobales	Methanotrichace	Methanothrix

Heimdallarchaeota	Methanofastidiosa		ae	
Candidatus Thorarchaeota	Thermoplasmata	Methanomicrobiales	Methanosarcinaceae	Pyrococcus
Candidatus Odinarchaeota	Methanobacteria	Methanomassiliicoccales	Methanoregulaceae	Methanomethylovorans
Candidatus Lokiarchaeota		Thermoproteales	Methanocorpusculaceae	Lokiarchaeum
Candidatus Bathyarchaeota		Methanocellales	Thermoproteaceae	Methanospirillum
Candidatus Diapherotrites		Methanobacteriales	Methanospirillaceae	Candidatus Methanofastidiosum
			Methanomassiliicoccaceae	Thermoproteus

				Methanocellaceae	Methanocorpusculum
				Methanobacteriaceae	Candidatus Nitrosomarinus
	Proteobacteria	Gammaproteobacteria	Enterobacterales	Enterobacteriaceae	Citrobacter
	Nitrospirae	Epsilonproteobacteria	Campylobacterales	Campylobacteraceae	Salmonella
	Aquificae	Nitrospira	Nitrospirales	Nitrospiraceae	Campylobacter
Bacteria	Firmicutes	Alphaproteobacteria	Aquificales	Acetobacteraceae	Nitrospira
	Bacteroidetes	Aquificae	Rhodospirillales	Aquificaceae	Hydrogenobacter
	Actinobacteria <actinobacteria>	Deltaproteobacteria	Acidithiobacillales	Acidithiobacillaceae	Acidiphilium
	Chloroflexi	Acidithiobacil	Myxococcales	Anaeromyxobact	Acidithiobacillus

			lia		eraceae	
			Clostridia	Alteromonadales	Shewanellaceae	Anaeromyxobacter
			Chitinophagia	Clostridiales	Geobacteraceae	Shewanella
			Betaproteobacteria	Chitinophagales	Clostridiales Family XVII. Incertae Sedis	Chitinophaga
			Dehalococcoidia	Desulfuromonadales	Chitinophagaceae	Sulfobacillus
			Actinobacteria			Geobacter
**Tetrathionate oxidation**	Archaea	Euryarchaeota	Halobacteria	Haloferacales	Haloferacaceae	Haloferax
		Crenarchaeota	Thermoprotei	Sulfolobales	Sulfolobaceae	Acidianus
		Candidatus Heimdallarchaeota	Thermoplasmata	Halobacteriales	Halobacteriaceae	Halalkalicoccus
		Thaumarchaeota	Candidatus	Thermoplasmat	Picrophilaceae	Picrophilus

		Poseidoniia	ales		
		Methanomicrobia	Candidatus Poseidoniales	Ferroplasmaceae	Acidiplasma
		Methanobacteria	Methanomicrobiales	Methanomicrobiaceae	Sulfolobus
			Methanobacteriales	Methanobacteriaceae	Methanoculleus
					Halarchaeum
					Methanobrevibacter
	Proteobacteria	Gammaproteobacteria	Pseudomonadales	Pseudomonadaceae	Pseudomonas
Bacteria	Firmicutes	Bacilli	Enterobacterales	Enterobacteriaceae	Bacillus
	Actinobacteria	Betaproteobac	Bacillales	Bacillaceae	Klebsiella

		<actinobacteria>	teria			
		Bacteroidetes	Actinobacteria	Burkholderiales	Vibrionaceae	Streptococcus
		Cyanobacteria	Alphaproteobacteria	Lactobacillales	Moraxellaceae	Escherichia
		Nitrospirae	Clostridia	Alteromonadales	Streptococcaceae	Vibrio
		Deinococcus-Thermus	Epsilonproteobacteria	Vibrionales	Burkholderiaceae	Acinetobacter
		Aquificae	Deltaproteobacteria	Oceanospirillales	Pasteurellaceae	Streptomyces
		Chloroflexi	Bacteroidia	Pasteurellales	Streptomycetaceae	Helicobacter
		Fibrobacteres	Nitrospira	Rhizobiales	Helicobacteraceae	Staphylococcus
**Tetrathionate**	Archaea	Euryarchaeota	Halobacteria	Haloferacales	Haloferacaceae	Haloferax

**reduction**	Thaumarchaeota<br>Candidatus Heimdallarchaeota<br>Candidatus Woesearchaeota<br>Candidatus Lokiarchaeota	Methanobacteria<br>Methanomicrobia<br>Nitrososphaeria<br>Archaeoglobi<br>Methanococci<br>Candidatus Poseidoniia	Methanobacteriales<br>Methanomicrobiales<br>Archaeoglobales<br>Methanococcales<br>Halobacteriales<br>Candidatus Nitrosocaldales<br>Candidatus Poseidoniales<br>Nitrososphaerales	Methanobacteriaceae<br>Methanomicrobiaceae<br>Methanococcaceae<br>Archaeoglobaceae<br>Halobacteriaceae<br>Candidatus Nitrosocaldaceae<br>Nitrososphaeraceae<br>Natrialbaceae	Methanothermobacter<br>Methanobrevibacter<br>Methanoculleus<br>Archaeoglobus<br>Methanococcus<br>Candidatus Caldiarchaeum<br>Methanobacterium<br>Halalkaliarchaeum

			Methanosarcinales	Halorubraceae	Candidatus Nitrosocaldus
			Natrialbales	Candidatus Methanoperedenaceae	Halalkalicoccus
					Candidatus Nitrosocosmicus
Bacteria	Proteobacteria	Gammaproteobacteria	Enterobacterales	Enterobacteriaceae	Salmonella
	Firmicutes	Bacilli	Pseudomonadales	Pseudomonadaceae	Pseudomonas
	Actinobacteria <actinobacteria>	Clostridia	Vibrionales	Vibrionaceae	Vibrio
	Bacteroidetes	Betaproteobacteria	Clostridiales	Yersiniaceae	Escherichia

		Fusobacteria	Actinobacteria	Burkholderiales	Streptococcaceae	Klebsiella
		Deinococcus-Thermus	Alphaproteobacteria	Bacillales	Clostridiaceae	Streptococcus
		Cyanobacteria	Epsilonproteobacteria	Lactobacillales	Aeromonadaceae	Clostridium
		Nitrospirae	Deltaproteobacteria	Aeromonadales	Bacillaceae	Citrobacter
		Planctomycetes	Flavobacteriia	Streptomycetales	Streptomycetaceae	Aeromonas
		Tenericutes	Fusobacteriia	Alteromonadales	Morganellaceae	Streptomyces
**Thiosulfate disproportionation**	Archaea	Euryarchaeota	Halobacteria	Halobacteriales	Haloarculaceae	Haloarcula
		Thaumarchaeota	Methanobacteria	Haloferacales	Haloferacaceae	Haloferax
		Candidatus	Nitrososphaeri	Methanobacteri	Methanobacteria	Halalkaliarchaeum

	Heimdallarchaeota	a	ales	ceae	
		Candidatus	Candidatus	Halobacteriaceae	Methanobrevibacte
		Poseidoniia	Poseidoniales		r
		Methanomicro	Candidatus	Candidatus	Halalkalicoccus
		bia	Nitrosocaldales	Nitrosocaldaceae	
			Methanomicrobi	Nitrososphaerace	Candidatus
			ales	ae	Nitrosocaldus
			Nitrososphaeral	Methanomicrobia	Candidatus
			es	ceae	Nitrosocosmicus
					Halarchaeum
					Methanoculleus
					Methanobacterium
	Proteobacteria	Gammaproteo	Enterobacterales	Enterobacteriace	Pseudomonas
Bacteria		bacteria		ae	
	Firmicutes	Bacilli	Pseudomonadal	Pseudomonadace	Escherichia

		es	ae	
Actinobacteria <actinobacteria>	Actinobacteria	Bacillales	Bacillaceae	Klebsiella
Bacteroidetes	Betaproteobacteria	Lactobacillales	Vibrionaceae	Bacillus
Spirochaetes	Alphaproteobacteria	Corynebacteriales	Streptococcaceae	Streptococcus
Deinococcus-Thermus	Clostridia	Burkholderiales	Streptomycetaceae	Vibrio
Cyanobacteria	Epsilonproteobacteria	Vibrionales	Burkholderiaceae	Streptomyces
Nitrospirae	Flavobacteriia	Streptomycetales	Mycobacteriaceae	Salmonella
Tenericutes	Bacteroidia	Rhizobiales	Staphylococcaceae	Staphylococcus

		Fusobacteria	Deltaproteobacteria	Alteromonadales	Helicobacteraceae	Helicobacter
**Thiosulfate oxidation**	Archaea	Euryarchaeota	Halobacteria	Haloferacales	Haloferacaceae	Haloferax
		Crenarchaeota	Thermoprotei	Sulfolobales	Sulfolobaceae	Acidianus
		Candidatus Heimdallarchaeota	Thermoplasmata	Thermoplasmatales	Halobacteriaceae	Halalkaliarchaeum
		Thaumarchaeota	Candidatus Poseidoniia	Halobacteriales	Picrophilaceae	Picrophilus
			Methanobacteria	Candidatus Poseidoniales	Ferroplasmaceae	Halalkalicoccus
			Methanomicrobia	Methanobacteriales	Methanomicrobiaceae	Methanoculleus
				Methanomicrobiales	Methanobacteriaceae	Acidiplasma
						Methanobrevibacte

					r
					Halarchaeum
					Sulfolobus
Bacteria	Proteobacteria	Gammaproteobacteria	Enterobacterales	Enterobacteriaceae	Pseudomonas
	Firmicutes	Alphaproteobacteria	Pseudomonadales	Pseudomonadaceae	Escherichia
	Actinobacteria <actinobacteria>	Bacilli	Bacillales	Bacillaceae	Bacillus
	Bacteroidetes	Betaproteobacteria	Burkholderiales	Burkholderiaceae	Klebsiella
	Deinococcus-Thermus	Actinobacteria	Rhizobiales	Bradyrhizobiaceae	Bradyrhizobium
	Cyanobacteria	Clostridia	Rhodobacterales	Rhodobacteraceae	Streptococcus

	Nitrospirae	Epsilonproteobacteria	Lactobacillales	Vibrionaceae	Salmonella
	Aquificae	Deltaproteobacteria	Alteromonadales	Moraxellaceae	Vibrio
	Chloroflexi	Deinococci	Vibrionales	Streptococcaceae	Acinetobacter
	Candidatus Rokubacteria	Bacteroidia	Oceanospirillales	Pasteurellaceae	Streptomyces
	Fibrobacteres				

Note: The top 10 abundant bacteria and archaea in each classification level were listed in the table.

Supplementary Table S4. The abundance of sulfur genes in in five habitats. UF, upland forest; DS, deep-sea sediments; MW, marine waters; RS, river sediments; MS, mangrove sediments.

Genes	UF	MW	MS	DS	RS
ssuB	0.78	1.62	1.06	1.17	1.69
ssuD	0.27	0.29	0.08	0.07	0.09
dmdB	0.23	0.35	0.27	0.25	0.38
cysC	0.26	0.33	0.21	0.29	0.22
sat	0.20	0.12	0.10	0.08	0.14
xsc	0.19	0.25	0.10	0.14	0.15
atsA	0.19	0.55	0.50	0.54	0.30
slcC	0.15	0.30	0.11	0.19	0.15
cysE	0.12	0.26	0.15	0.20	0.24
hdrA	0.13	0.18	0.71	0.90	0.32
dmsA	0.14	0.10	0.24	0.21	0.23
cysK	0.09	0.15	0.11	0.17	0.14
tusA	0.09	0.18	0.16	0.24	0.16
SUOX	0.09	0.03	0.04	0.03	0.03
ttrB	0.08	0.05	0.16	0.08	0.10
cysN	0.12	0.16	0.12	0.12	0.15
glpE	0.07	0.19	0.09	0.04	0.12
tauD	0.08	0.31	0.06	0.07	0.07
cysJ	0.07	0.10	0.06	0.08	0.14
metB	0.07	0.19	0.08	0.08	0.15
sorA	0.04	0.02	0.01	0.01	0.01
dddP	0.05	0.05	0.02	0.02	0.01
ssuC	0.04	0.08	0.03	0.03	0.06
MET3	0.03	0.01	0.01	0.02	0.01
hdrD	0.03	0.06	0.17	0.19	0.09
cysH	0.04	0.09	0.04	0.04	0.07
sir	0.03	0.03	0.03	0.02	0.08
dmdA	0.04	0.35	0.04	0.03	0.05
cysQ	0.03	0.25	0.09	0.09	0.15
sseA	0.02	0.09	0.02	0.01	0.04
ssuE	0.03	0.02	0.02	0.03	0.03
SoxC	0.03	0.01	0.00	0.00	0.01
msmA	0.03	0.08	0.02	0.00	0.02
ssuA	0.02	0.04	0.03	0.03	0.06

comE	0.03	0.05	0.02	0.02	0.02
dmdC	0.02	0.05	0.03	0.01	0.08
tmoC	0.01	0.03	0.00	0.01	0.01
nrnA	0.02	0.04	0.07	0.10	0.05
fccB	0.01	0.02	0.03	0.01	0.05
cysNC	0.02	0.02	0.02	0.02	0.03
apt	0.01	0.00	0.00	0.01	0.00
phsB	0.01	0.00	0.02	0.02	0.01
asrB	0.02	0.02	0.04	0.11	0.05
sqr	0.01	0.01	0.02	0.01	0.04
aprA	0.02	0.07	0.10	0.03	0.02
cysI	0.02	0.05	0.02	0.02	0.03
ttrA	0.02	0.01	0.02	0.01	0.03
msuD	0.01	0.01	0.00	0.00	0.00
tsdB	0.01	0.02	0.01	0.00	0.03
dddD	0.01	0.03	0.02	0.02	0.03
SELENBP1	0.01	0.01	0.00	0.00	0.00
MET17	0.01	0.04	0.01	0.01	0.02
pspE	0.01	0.03	0.05	0.03	0.04
ETHE1	0.01	0.06	0.02	0.02	0.02
mccB	0.01	0.01	0.01	0.01	0.01
slcD	0.01	0.02	0.00	0.00	0.01
hdrC	0.01	0.00	0.06	0.09	0.03
soxV	0.01	0.03	0.01	0.03	0.01
cysD	0.01	0.02	0.02	0.01	0.04
tsdA	0.01	0.00	0.00	0.00	0.01
suyB	0.01	0.05	0.01	0.02	0.03
dmdD	0.01	0.04	0.02	0.02	0.03
shyC	0.01	0.02	0.01	0.02	0.01
soxB	0.01	0.02	0.03	0.02	0.04
soxD	0.00	0.02	0.01	0.00	0.02
asrC	0.01	0.01	0.04	0.10	0.02
ddhA	0.01	0.00	0.07	0.05	0.02
npsr	0.00	0.00	0.01	0.01	0.01
doxD	0.00	0.00	0.00	0.00	0.00
dmsB	0.00	0.00	0.02	0.01	0.01
cuyA	0.01	0.03	0.01	0.02	0.01
SOX	0.00	0.00	0.00	0.00	0.00
MET5	0.00	0.00	0.01	0.01	0.01
hydG	0.00	0.04	0.00	0.02	0.01
dsrP	0.00	0.00	0.04	0.01	0.01
sorB	0.00	0.02	0.01	0.00	0.02

comC	0.01	0.02	0.00	0.03	0.01
aprB	0.01	0.02	0.02	0.00	0.00
mddA	0.00	0.00	0.01	0.00	0.00
Tmm	0.00	0.04	0.00	0.00	0.00
MET10	0.00	0.00	0.01	0.01	0.01
MET22	0.00	0.00	0.00	0.00	0.00
dddW	0.00	0.01	0.00	0.00	0.00
sdo	0.00	0.01	0.01	0.02	0.01
dsoF	0.00	0.01	0.01	0.00	0.01
dsrF	0.00	0.00	0.03	0.00	0.01
sreA	0.00	0.00	0.00	0.00	0.00
mtsA	0.00	0.01	0.02	0.04	0.01
comD	0.00	0.01	0.00	0.00	0.00
dsrN	0.00	0.02	0.02	0.01	0.01
mtsB	0.00	0.00	0.02	0.04	0.01
ttrC	0.00	0.00	0.00	0.00	0.00
rhd	0.00	0.00	0.00	0.00	0.00
tetH	0.00	0.00	0.00	0.00	0.00
tmoF	0.00	0.00	0.00	0.00	0.00
dsrO	0.00	0.00	0.01	0.00	0.00
dmsC	0.00	0.00	0.00	0.00	0.01
sor	0.00	0.00	0.00	0.00	0.00
fccA	0.00	0.00	0.01	0.00	0.01
phsC	0.00	0.00	0.01	0.00	0.01
SoeA	0.00	0.00	0.00	0.00	0.00
hydD	0.00	0.00	0.01	0.02	0.01
ddhB	0.00	0.00	0.00	0.00	0.00
dsrB	0.00	0.00	0.05	0.05	0.02
psrB	0.00	0.00	0.00	0.00	0.00
hydA	0.00	0.00	0.01	0.04	0.03
soxY	0.00	0.00	0.01	0.00	0.01
phsA	0.00	0.00	0.01	0.01	0.01
rdlA	0.00	0.00	0.00	0.00	0.00
suyA	0.00	0.01	0.00	0.00	0.00
dsrA	0.00	0.00	0.04	0.01	0.02
asrA	0.00	0.00	0.02	0.03	0.01
dsrK	0.00	0.01	0.10	0.02	0.03
soxX	0.00	0.00	0.01	0.00	0.00
hdrE	0.00	0.00	0.02	0.01	0.00
hdrB	0.00	0.00	0.02	0.01	0.01
TST	0.00	0.00	0.00	0.00	0.00
otr	0.00	0.00	0.02	0.00	0.02
dsoD	0.00	0.00	0.00	0.00	0.01
dddY	0.00	0.00	0.00	0.00	0.00

dddQ	0.00	0.01	0.00	0.00	0.00
dmoB	0.00	0.00	0.00	0.01	0.00
soxZ	0.00	0.00	0.00	0.00	0.00
soxA	0.00	0.00	0.01	0.00	0.01
sirA	0.00	0.00	0.00	0.00	0.00
hdrF	0.00	0.00	0.00	0.00	0.00
qmoA	0.00	0.00	0.00	0.00	0.00
SoeB	0.00	0.00	0.00	0.00	0.00
mccA	0.00	0.00	0.00	0.00	0.00
APR	0.00	0.00	0.00	0.00	0.00
sorT	0.00	0.00	0.00	0.00	0.00
dsrC	0.00	0.00	0.02	0.01	0.01
soxW	0.00	0.00	0.00	0.00	0.00
ATCYSC1	0.00	0.00	0.00	0.00	0.00
shyB	0.00	0.00	0.00	0.01	0.00
dsrD	0.00	0.00	0.00	0.00	0.00
PAPSS	0.00	0.00	0.00	0.00	0.00
SQOR	0.00	0.00	0.00	0.00	0.00
dsrH	0.00	0.00	0.00	0.00	0.00
ddhC	0.00	0.00	0.00	0.00	0.00
MPST	0.00	0.00	0.00	0.00	0.00
soeC	0.00	0.00	0.00	0.00	0.00
HINT4	0.00	0.00	0.00	0.00	0.00
dmoA	0.00	0.00	0.00	0.00	0.00
atsK	0.00	0.00	0.00	0.00	0.00
psrA	0.00	0.00	0.03	0.01	0.01
fsr	0.00	0.00	0.00	0.01	0.00
psrC	0.00	0.00	0.00	0.00	0.00
dsrM	0.00	0.00	0.02	0.01	0.01
RDL	0.00	0.00	0.00	0.00	0.00
dsoC	0.00	0.00	0.00	0.00	0.00
sfnG	0.00	0.00	0.00	0.00	0.00
APA1_2	0.00	0.00	0.00	0.00	0.00
dsrJ	0.00	0.00	0.00	0.00	0.00
sreB	0.00	0.00	0.00	0.00	0.00
aprM	0.00	0.01	0.00	0.00	0.00
ygaP	0.00	0.00	0.00	0.00	0.00
msmB	0.00	0.00	0.00	0.00	0.00
soxS	0.00	0.00	0.00	0.00	0.00
SAL	0.00	0.00	0.00	0.00	0.00
dsoE	0.00	0.00	0.00	0.00	0.00
soxl	0.00	0.00	0.00	0.00	0.00
hydB	0.00	0.00	0.00	0.00	0.00

dddL	0.00	0.00	0.00	0.00	0.00
dsrE	0.00	0.00	0.00	0.00	0.00
dsoB	0.00	0.00	0.00	0.00	0.00
BPNT1	0.00	0.00	0.00	0.00	0.00
IMPAD1	0.00	0.00	0.00	0.00	0.00

Supplementary Figure S1. Overview of **(a)** assimilatory sulfate reduction and **(b)** dissimilatory sulfate reduction pathway and related genes. APS: adenosine 5'-phosphosulfate; PAPS: phosphoadenosine 5'-phosphosulfate.

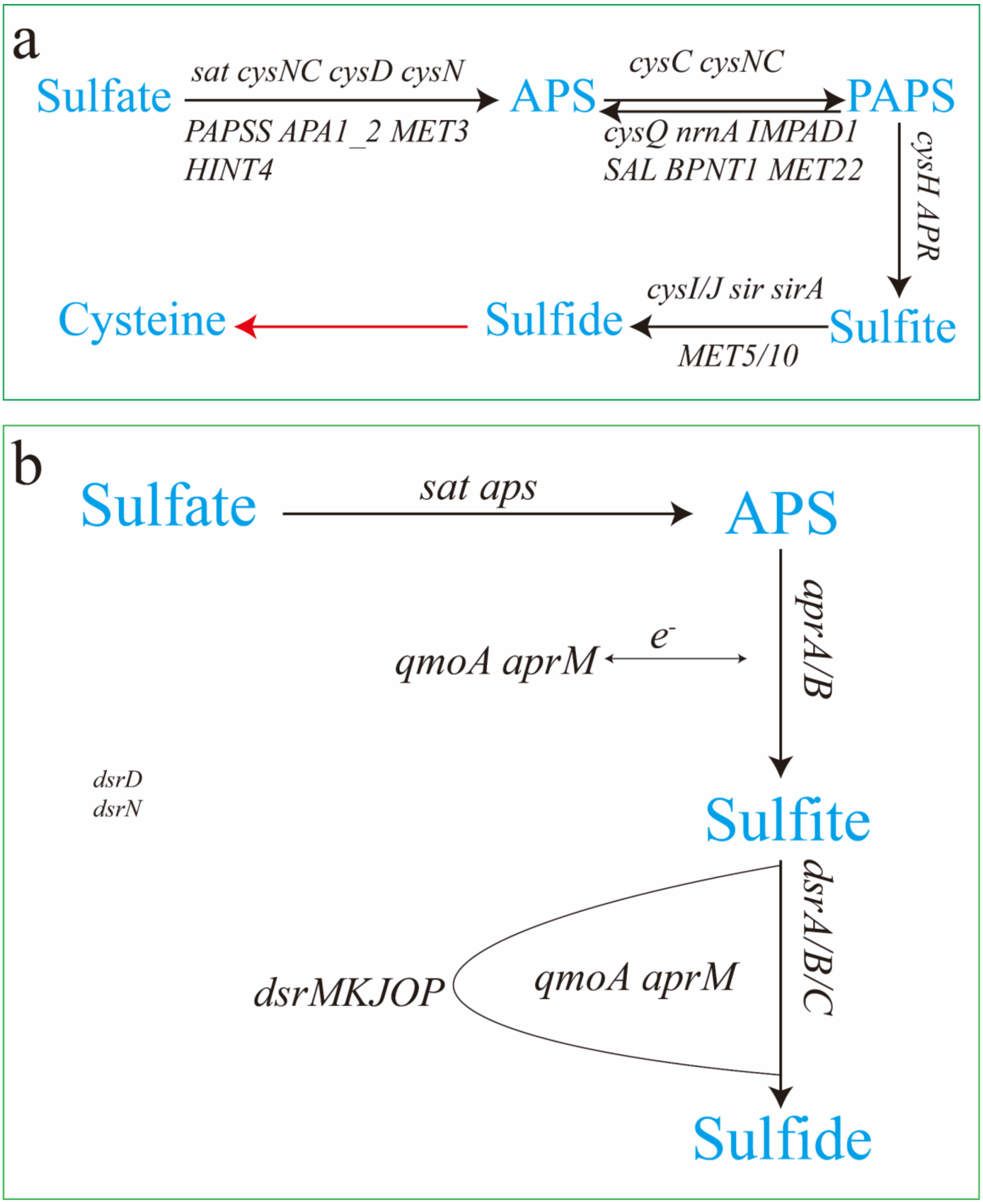

Supplementary Figure S2. Overview of organic degradation and synthesis pathway and related genes. DMSP: dimethylsulfoniopropionate; DMS: dimethyl sulfide; MeSH: methanethiol; MMPA: methylmercaptopropionate; DMSO: sulfoxide.

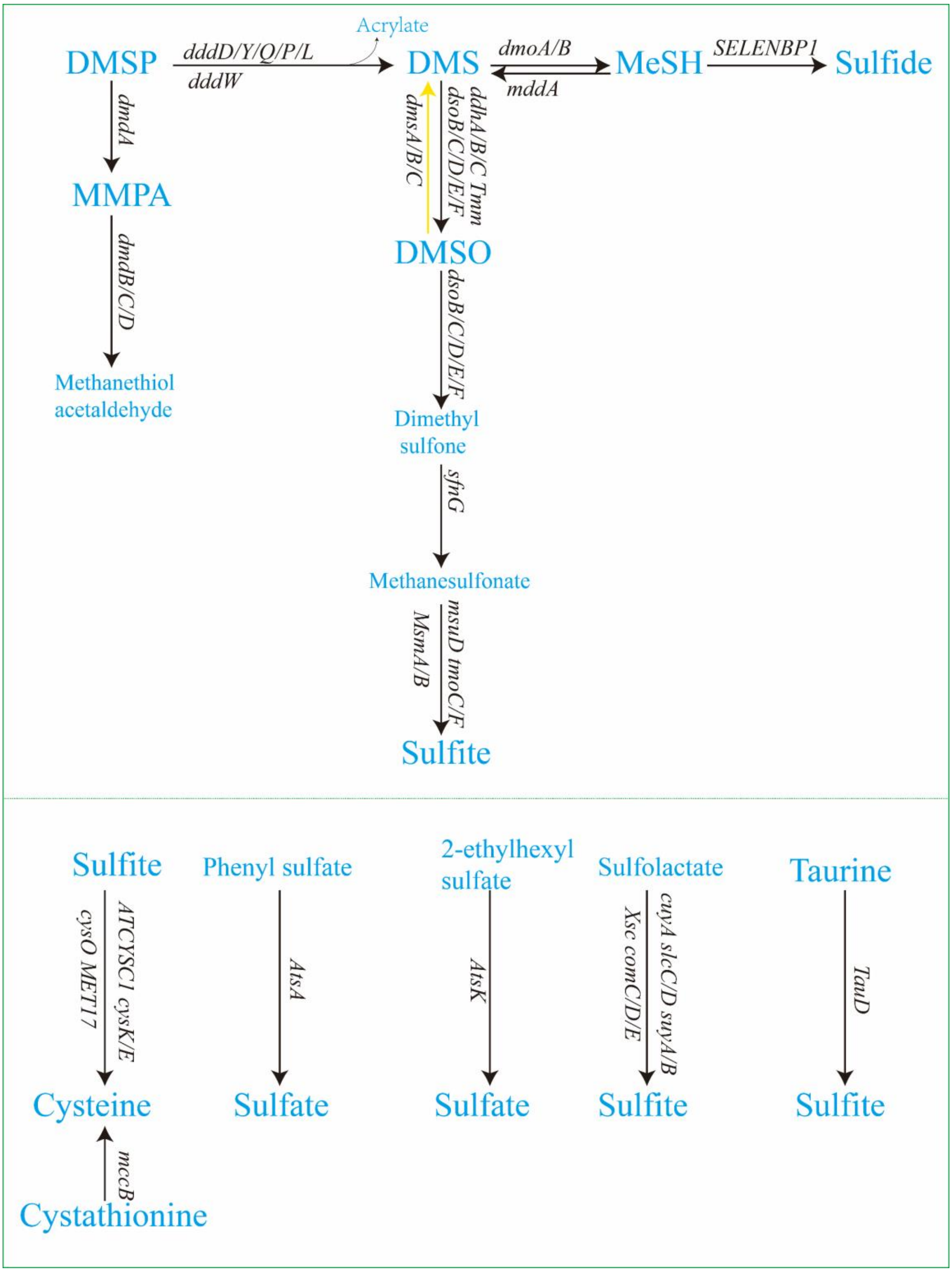

Supplementary Figure S3. Overview of **(a)** **s**ulfide oxidation and **(b)** **s**ulfi**t**e oxidation pathway and related genes.

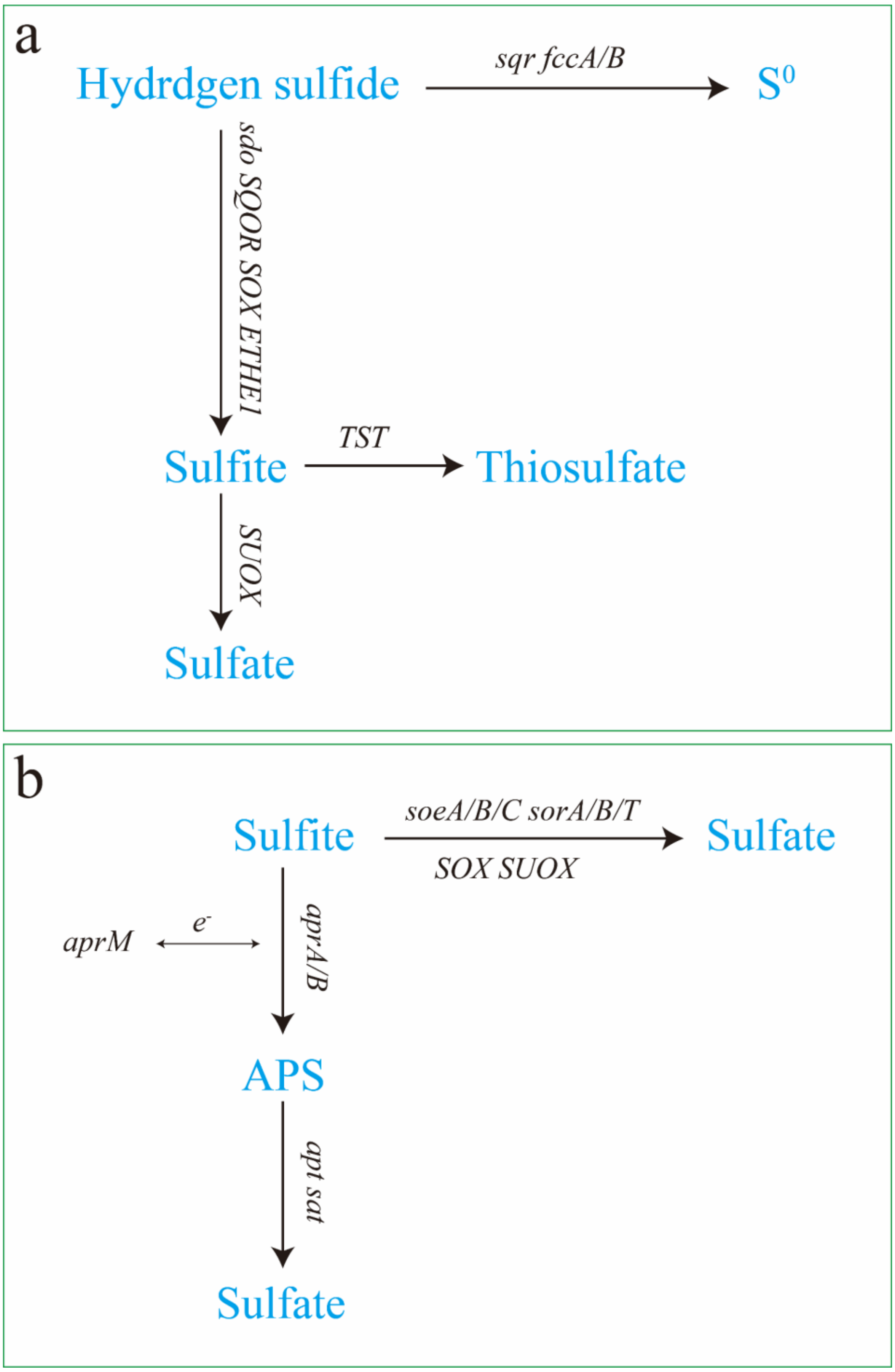

Supplementary Figure S4. Overview of (a) sulfur oxidation and (b) sulfur reduction pathway and related genes.

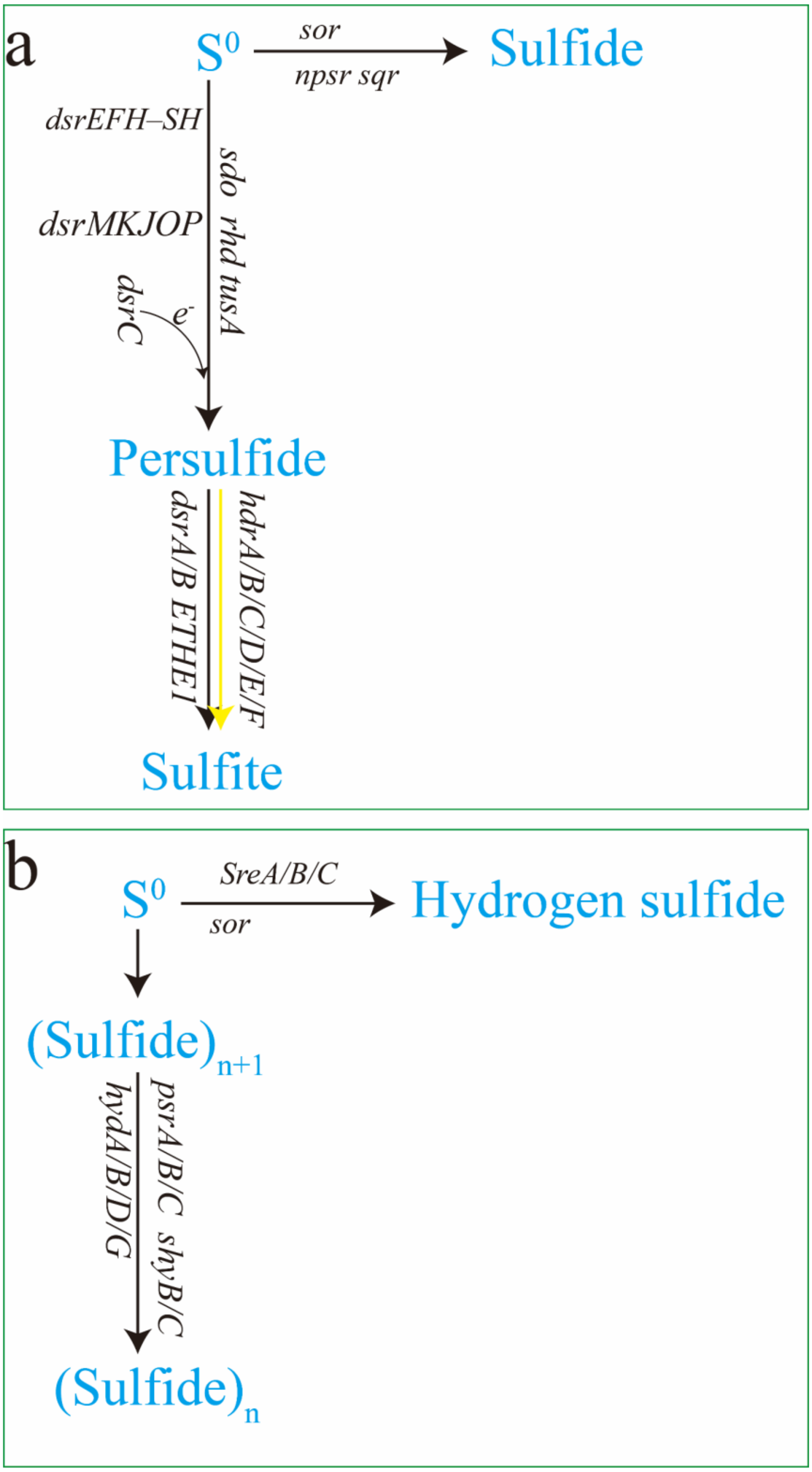

Supplementary Figure S5. Overview of **(a)** tetrathionate oxidation, **(b)** tetrathionate reduction, **(c)** thiosulfate disproportionation, and **(d)** thiosulfate oxidation pathway and related genes.

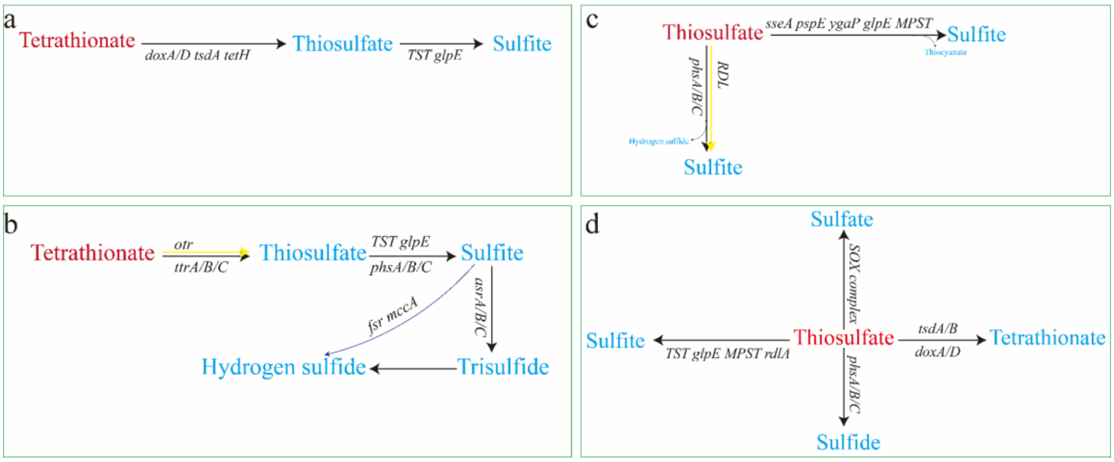

Supplementary Figure S6. Sulfur cycle genes detected using the SMDB, NR, M5nr, KEGG, eggNOG, COG databases.

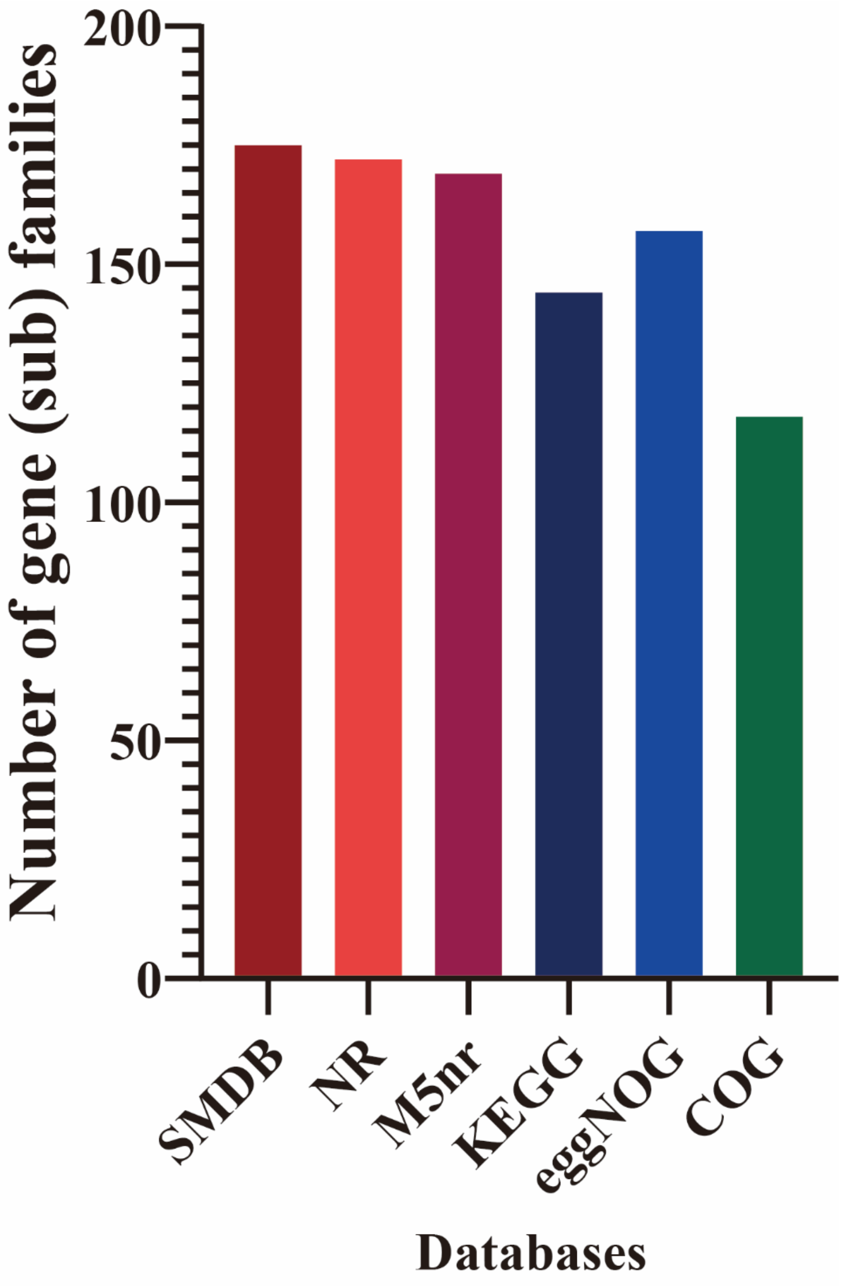

Supplementary Figure S7. Phylogenetic tree of *phsA* and *psrA* sequences present in the SMDB. Red label color represents *phsA* sequences, blue label color represents *psrA* sequences.

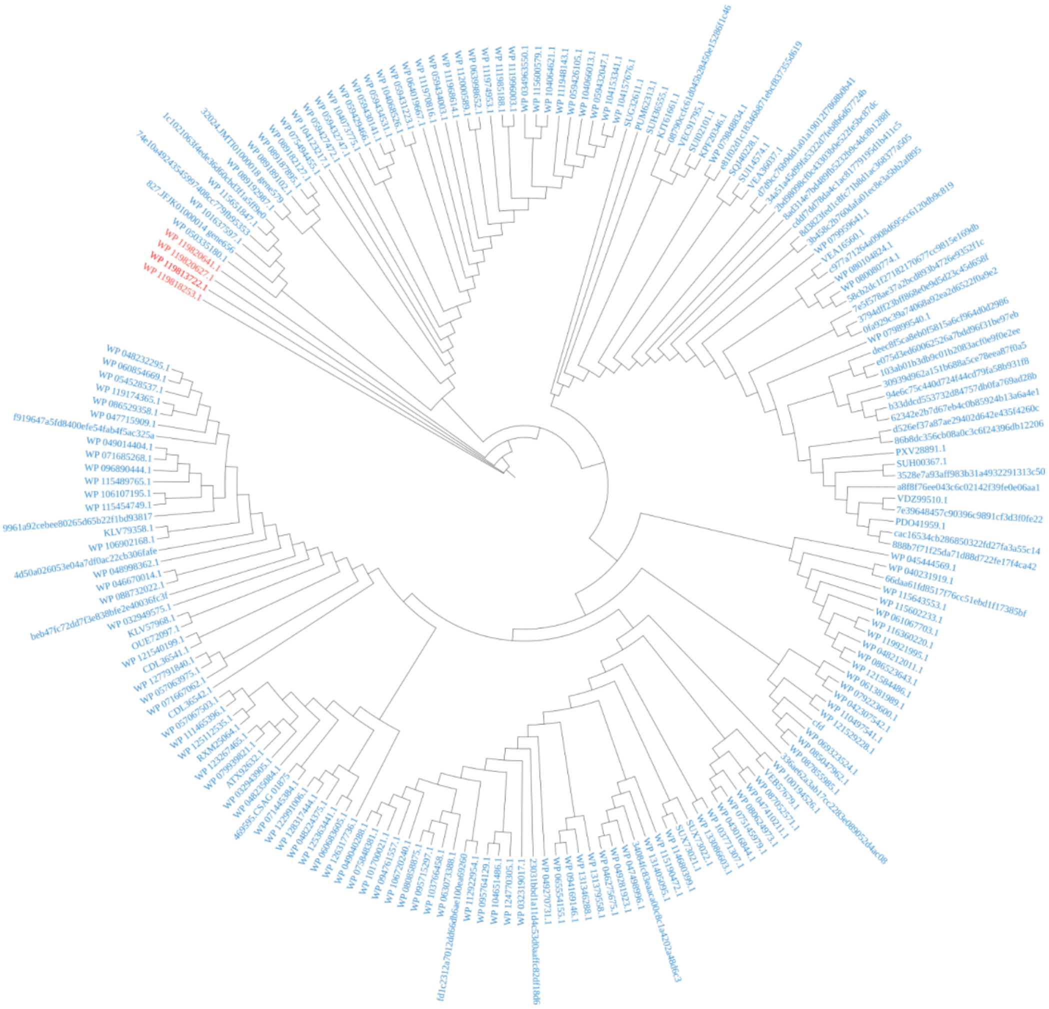

Supplementary Figure S8. Taxonomic composition of microorganisms at the class level. Different colors refer to different class. UF, upland forest; DS, deep-sea sediments; MW, marine waters; RS, river sediments; MS, mangrove sediments.

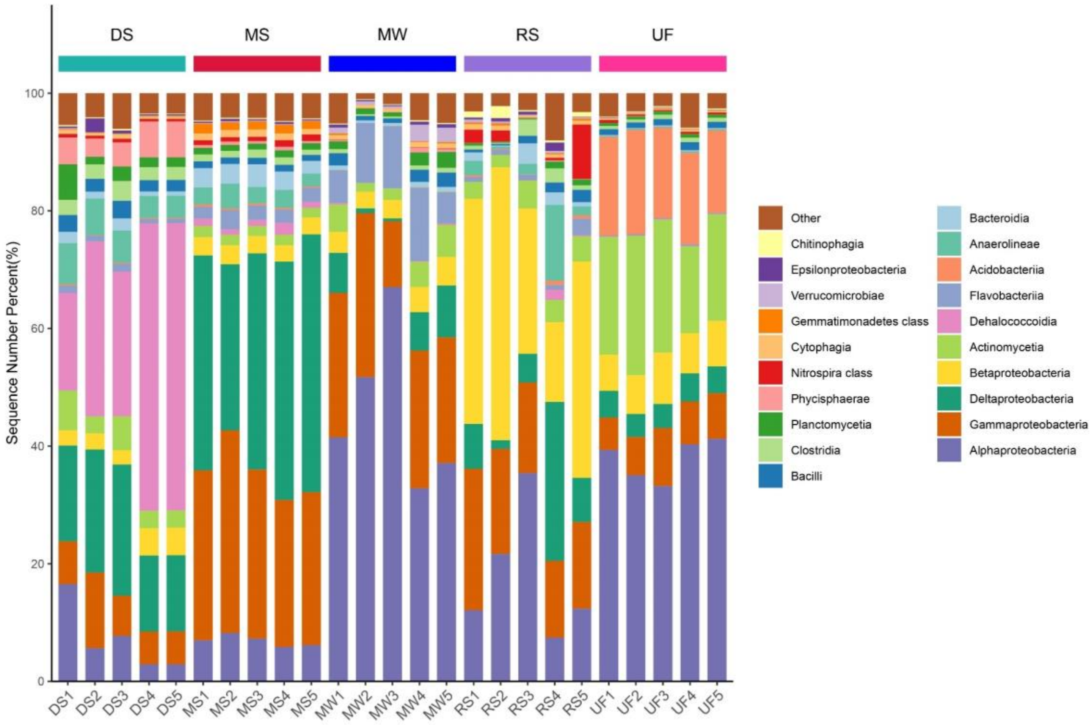

Supplementary Figure S9. Taxonomic composition of sulfur metabolizing microorganisms at the phylum level. Different colors refer to different class. UF, upland forest; DS, deep-sea sediments; MW, marine waters; RS, river sediments; MS, mangrove sediments.

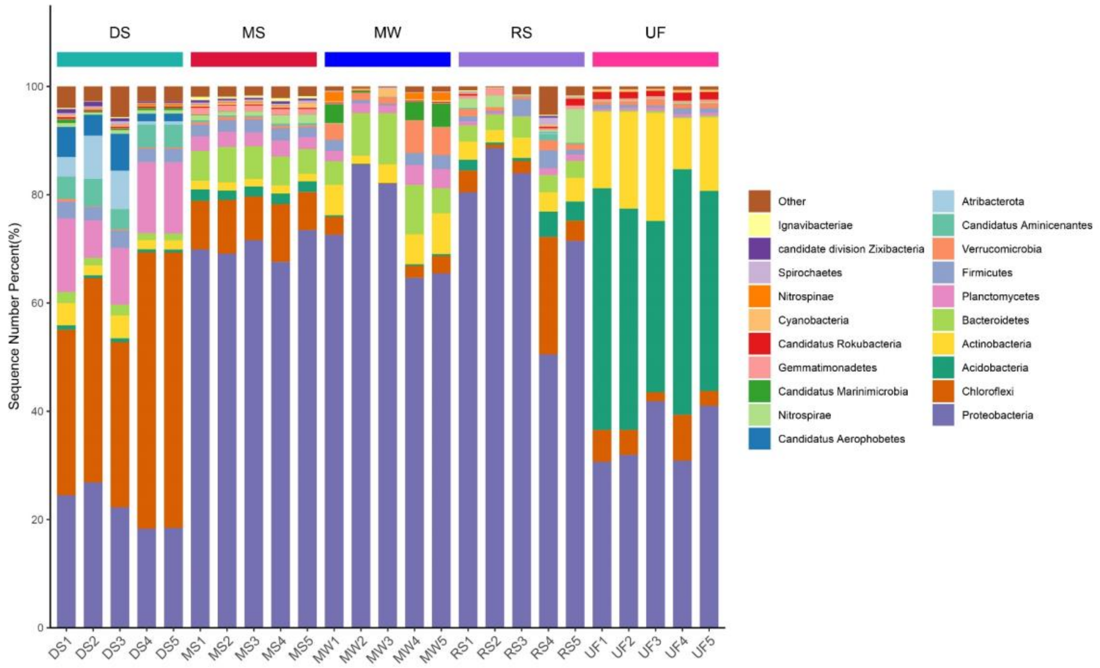

Summary of the sulfur metabolism pathway genes

Assimilatory sulfate reduction

Assimilatory sulfate reduction, catalyzing the reduction of sulfate to sulfide for the purpose of biosynthetic. An essential step in ASR is the same as in DSR, that is, sulfate activation in reaction with ATP. A total of 26 sulfur genes with 220,757 sequences and 144,485 homologous sequences were included in this pathway (Table 1, Supplementary Table S2, Figure S1A). The enzymes encoded by *cysNC, cysN, cysD, PAPSS, APA1_2, MET3, HINT4*, and *sat* converted sulfate to adenosine 5′-phosphosulphate (APS), and *cysC, nrnA, cysQ, IMPAD1, MET22*, and *SAL* converted APS to phosphoadenosine 5′-phosphosulphate (PAPS). *CysH* and *APR* proteins converted PAPS to sulfite. *CysI/J, sir, sirA, MET10,* and *MET5* proteins catalyzed the reduction of sulfite to sulfide. *HINT4* encodes the biofunctional enzyme that responsible for sulfate conversion to AMP.

Dissimilatory sulfate reduction

Dissimilatory sulfate reduction, sulfate reduction for the purpose of energy production in the absence of oxygen environments. A total of 33 sulfur genes with 257,327 sequences and 153,717 homologous sequences were included in this pathway (Table 1, Supplementary Table S2, Figure S1B). The enzymes encoded by sat converted APS to APS. *AprA/B, aprM*, and *qmoA* proteins catalyzed reversibly the reduction of APS to sulfite and AMP during dissimilatory sulfate reduction. *DsrA/B/C* protein catalyzed the reduction of sulfite to sulfide. *DsrM/K/J/O/P* was a sulfite reduction-associated complex DsrMKJOP protein.

Organic degradation/synthesis

A total of 57 sulfur genes with 294,282 sequences and 250,357 homologous sequences were included in this pathway (Table 1, Supplementary Table S2, Figure S2). The enzymes encoded by *ATCYSC1, cysK/E, cysO*, and *MET17* catalyzed sulfide synthesis to L-cysteine. The gene *atsA* encodes arylsulfatase catalyzed the reduction of phenyl sulfate to sulfate. The gene *atsK* encodes alpha-ketoglutarate-dependent sulfate ester dioxygenase catalyzed the reduction of 2-ethylhexyl sulfate to sulfate. *Xsc, comC/D/E, cuyA, slcC/D*, and *suyA/B* proteins were involved in sulfolactate degradation. *DddD/L/P/Q/W/Y* encoded DMSP lyase for the conversion of DMSP to DMS and acrylate, and *dmdA/B/C/D* was involved in the conversion of DMSP to methanethiol acetaldehyde. The enzymes encoded by *dmsA/B/C, ddhA/B/C, dsoB/C/D/E/F*, and *Tmm* were involved in the transformation between DMS and sulfoxide (DMSO). *DmoA/B, MddA, sfnG*, and *SELENBP1* proteins were involved in the conversion of MeSH. *MsmA/B, tmoC/F*, and *msuD* proteins were involved in the conversion of methanesulfonate to sulfite. *MtsA/B* protein encodes coenzyme M methyltransferase was involved in methanogenesis from methylated thiols. *SsuA/B/C/D/E* was a sulfonate transport system of substrate-binding proteins. The gene *tauD* encodes taurine dioxygenase was involved in the degradation of taurine to sulfite. The gene *MccB* encodes cystathionine gamma-lyase catalyzed the conversion of cystathionine to cysteine and homocysteine to sulfide.

Sulfide oxidation

A total of nine sulfur genes with 8,990 sequences and 7,974 homologous

sequences were included in this pathway (Table 1, Supplementary Table S2, Figure S3A). The enzymes encoded by *fccA/B*, and *sqr* catalyzed the oxidation of sulfide to S^0. The enzymes encoded by *ETHE1, sdo, SQOR*, and *SOX* catalyzed the oxidation of sulfide to sulfite. Then, *SUOX* and *TST* protein catalyzed the oxidation of sulfite to sulfate and thiosulfate, respectively.

Sulfite oxidation

A total of 13 sulfur genes with 60,318 sequences and 39,043 homologous sequences were included in this pathway (Table 1, Supplementary Table S2, Figure S3B). These proteins catalyzed the oxidation of sulfite to sulfate.

Sulfur oxidation

A total of 27 sulfur genes with 76,367 sequences and 55,066 homologous sequences were included in this pathway (Table 1, Supplementary Table S2, Figure S4A). The sulfur atoms of elemental sulfur were transferred to sulfite reductase via *tusA*, *rhd, dsrEFH,* and *dsrC* proteins and then oxidized to sulfite. *DsrC* protein belongs to the *DsrC/TusE/RpsA* superfamily, and contains two highly conserved cysteine residues: DsrC-Cys100 and DsrC-Cys111. The 2 active Cys of *DsrC* protein and the received sulfur atoms form a persulfide under the catalysis of membrane binding protein complex DsrMKJOP, and finally the sulfite is produced under the catalysis of *DsrAB* protein. *ETHE 1*, and *hdrA/B/C/D/E/F* proteins were involved in the conversion of persulfide to sulfite. *Sdo* protein was involved in the conversion of S^0 to sulfite. *Npsr* and *sqr* proteins were involved in the transformation between S^0 and sulfide.

Sulfur reduction

A total of 13 sulfur genes with 953 sequences and 814 homologous sequences were included in this pathway (Table 1, Supplementary Table S2, Figure S4B). The enzymes encoded by *hydA/B/D/G, psrA/B/C*, and *shyB/C* were involved in the conversion of S^0 to polysulfide. *SreA/B/C* protein was involved in the conversion of S^0 to sulfide.

Tetrathionate oxidation

A total of 6 sulfur genes with 59,381 sequences and 38,309 homologous sequences were included in this pathway (Table 1, Supplementary Table S2, Figure S5A). The enzymes encoded by *doxA/D, tsdA,* and *tetH* were involved in the conversion of tetrathionate to thiosulfate. Then, thiosulfate was catalyzed to produce sulfite via *TST* and *glpE* proteins.

Tetrathionate reduction

A total of 12 sulfur genes with 50,634 sequences and 11,263 homologous sequences were included in this pathway (Table 1, Supplementary Table S2, Figure S5B). The enzymes encoded by *ttrA/B/C*, and *otr* were involved in the reduction of tetrathionate to thiosulfate. Then, thiosulfate was catalyzed to produce sulfide via *phsA/B/C* and *asrA/B/C* proteins.

Thiosulfate disproportionation

A total of 9 sulfur genes with 47,967 sequences and 36,351 homologous sequences were included in this pathway (Table 1, Supplementary Table S2, Figure S5C). These proteins were involved in the conversion of thiosulfate to sulfite.

Thiosulfate oxidation

A total of 21 sulfur genes with 43,398 sequences and 31,078 homologous

sequences were included in this pathway (Table 1, Supplementary Table S2, Figure S5D). The enzymes encoded by *tsdA/B* and *doxA/D* were involved in the conversion of thiosulfate to tetrathionate. *TST, glpE, MPST*, and *rdlA* proteins were involved in the conversion of thiosulfate to sulfite. *PhsA/B/C* protein was involved in the conversion of thiosulfate to sulfide. The SOX complex was involved in the conversion of thiosulfate to sulfate.
